# Supplementary material for: Critical impacts of interfacial water on C–H activation in photocatalytic methane conversion
Source: Commun Chem. 2023 Jan 20;6:8. doi: 10.1038/s42004-022-00803-3 (PMC9860031; doi:10.1038/s42004-022-00803-3)
Supplement: Supplementary file 2 — Supplementary Inforamtion [file 42004_2022_803_MOESM2_ESM.pdf]

Supplementary Information

Critical impacts of interfacial water on C–H activation in photocatalytic methane conversion

Hiromasa Sato[1,2], Atsushi Ishikawa[3], Hikaru Saito[1], Taisuke Higashi[1], Kotaro Takeyasu[4] and Toshiki Sugimoto[1,2,5]*

[1]Department of Materials Molecular Science, Institute for Molecular Science, Okazaki, Aichi 444-8585, Japan
[2]The Graduate University for Advanced Studies, SOKENDAI, Hayama, Kanagawa 240-0193, Japan
[3]Center for Green Research on Energy and Environmental Materials (GREEN), National Institute for Materials Science (NIMS), Tsukuba, Ibaraki 305-0044, Japan
[4]Faculty of Pure and Applied Sciences, Tsukuba Research Centre for Energy and Materials Science, University of Tsukuba, Tsukuba, Ibaraki, 305-8573 Japan
[5]Precursory Research for Embryonic Science and Technology (PRESTO), Japan Science and Technology Agency (JST), Chiyoda, Tokyo 102-0076, Japan
*e-mail: toshiki-sugimoto@ims.ac.jp

Contents:

Supplementary Notes

1. Evaluation of adsorbed water by DRIFT spectroscopy.

2. Supplementary data of photocatalytic reaction.

3. IR spectra of HDO molecules on photocatalyst surfaces.

4. SEM and TEM images of the photocatalyst samples.

5. MD simulation on the photocatalytic pre-activation of interfacial water under wet reaction condition.

6. Kinetic analysis for derivation of methane conversion rates.

7. IR absorption spectroscopy in the C–H stretching region.

8. Adsorption features of CH_4 without UV irradiation.

9. Effects of sample heating on photocatalysis.

Supplementary References

Supplementary Note 1

Evaluation of adsorbed water by DRIFT spectroscopy.

Excitation by UV irradiation increased the temperature of the photocatalyst surface by approximately 20 K under our experimental conditions (*c.f.* Methods). Owing to the temperature increase upon UV irradiation and the resultant thermal desorption, the adsorbed water molecules are partially released as water vapor. To quantitatively estimate the amount of water molecules adsorbed under photocatalytic reaction conditions, we conducted DRIFT spectroscopy of the water adsorbed on the sample photocatalyst surfaces as a function of relative humidity (RH). Figs. S1-1a and S1-1b show the DRIFT spectra of the adsorbed water molecules at room temperature (~295 K). The bending mode band at approximately 1600 cm^{-1} and the stretching mode band at approximately 3400 cm^{-1} increased in intensity with increasing water vapor pressure. In our previous study[1], we demonstrated that the peak area of the IR absorption derived from adsorbed water molecules (particularly the bending mode at approximately 1600 cm^{-1}) is proportional to the amount of adsorbed water molecules. The areas of these peaks were plotted as a function of water vapor pressure, as shown in Figs. S1-1c and S1-1d. The peak area (A) was fitted by the following Brunauer–Emmett–Teller (BET) adsorption isotherm[2]

$$A = a\frac{cx}{(1-x)(1-x+cx)}, \qquad (S1\_1)$$

where a is the peak area derived from one monolayer (ML), which corresponds to the number of water molecules in the order of ~1×10^{15} molecules/cm^2.[1] BET constant (c) and relative humidity (x) are defined as follows.

$$c \equiv K_\mathrm{c} \exp\left(\frac{E_1 - E_\mathrm{M}}{k_\mathrm{B}T_\mathrm{s}}\right), \qquad (S1\_2)$$

$$x \equiv \frac{P_{\mathrm{H_2O}}}{P^{\ominus}_{\mathrm{H_2O}}(T_\mathrm{s})}, \qquad (S1\_3)$$

where E_1 and E_M are the adsorption energies for the first layer and the multilayer, respectively, k_B and T_s are the Boltzmann constant and temperature of the photocatalyst surface, respectively. K_c $\left(\equiv \frac{k_\mathrm{dM}}{k_\mathrm{aM}}\frac{k_\mathrm{a1}}{k_\mathrm{d1}}\right)$ is usually assumed to be unity[2,3] and is defined by *pre-exponential factors* of the adsorption rate constant for the first layer (k_a1), the adsorption rate constant for the multilayer (k_aM), the desorption rate constant for the first layer (k_d1), and the desorption rate constant for the multilayer (k_dM). Relative humidity is defined as the partial pressure of water vapor ($P_{\mathrm{H_2O}}$) in a gas environment over the saturation pressure of water vapor ($P_{\mathrm{H_2O}}^{\ominus}$) at the surface temperature (T_s).

As shown in Figs. S1-1c and S1-1d, P_{H_2O} profiles of the water adsorption were well fitted with the BET adsorption isotherm with the parameters listed in Table S1-1. Under UV irradiation at P_{H_2O} = 2 kPa, the surface temperature increased from ~295 to ~318 K. Based on the saturation vapor pressure ($P_{H_2O}^{\ominus}$) at 318 K, the relative humidity (RH) at P_{H_2O} = 2 kPa was estimated to be 21%. The blue lines in Figs. S1-1c and S1-1d indicate the amount of adsorbed water layers covering the Pt/Ga_2O_3 and Pt/$NaTaO_3$ samples under the reaction conditions at 21% RH, which are 1.1 ML and 1.2 ML, respectively. Notably, the decrease in adsorbed water molecules during the reaction is at most 4%, and thus negligible, which is estimated from the maximum CO_2 production yields in our photocatalytic activity evaluation.

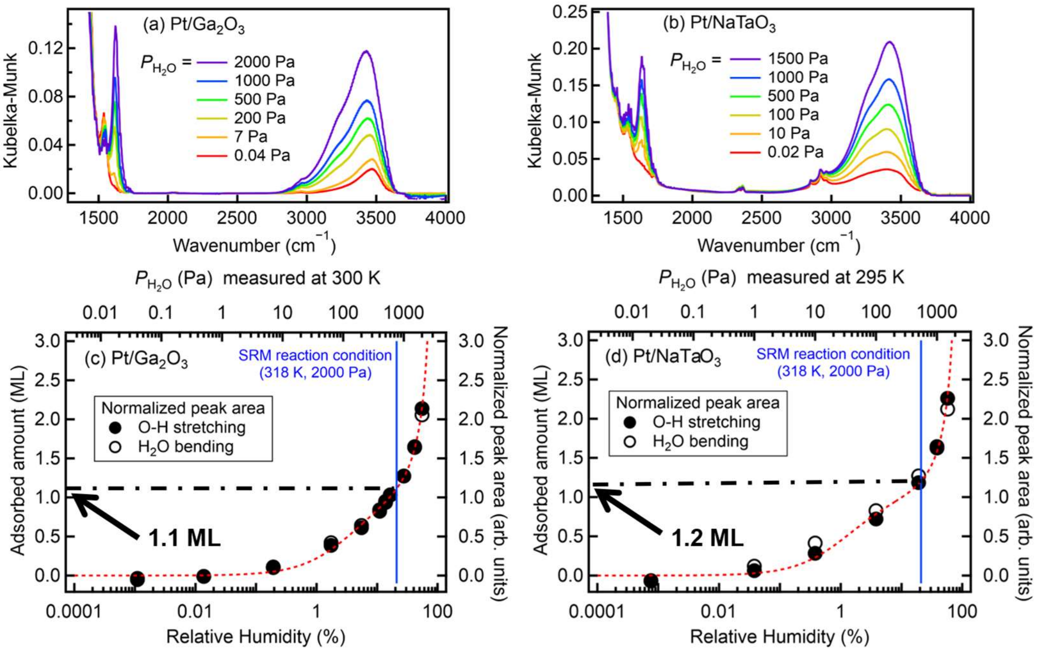

Figure S1-1. IR spectra of water species adsorbed on (a) Pt/Ga_2O_3 and (b) Pt/$NaTaO_3$ photocatalyst surfaces at various water vapor pressures (P_{H_2O}). The background spectra were obtained using Si powder in diffuse reflectance mode. The measured IR peak areas normalized by the fitting parameter *a* for both the O–H stretching mode and H–O–H bending mode as a function of relative humidity (RH) for (c) Pt/Ga_2O_3 and (d) Pt/$NaTaO_3$ photocatalysts. Red dotted line shows the RH dependence of the amount of adsorbed water molecules in units of ML (~1×10^{15} molecules/cm^2) derived from a curve fitting with the BET adsorption isotherm[1]. The blue vertical lines represent the RH (21%) under the actual photocatalytic reaction conditions with UV irradiation (P_{H_2O} = 2000 Pa and T_s = 318 K), indicating that 1.1 ML and 1.2 ML water molecules are adsorbed on Pt/Ga_2O_3 and Pt/$NaTaO_3$ samples under the reaction conditions.

Table S1-1. Fitting parameters of adsorbed water by the BET isotherm.

	$E_1 - E_M$ (kJ/mol)	E_1 (kJ/mol) [a]	T_s (K)
Pt/Ga_2O_3	8.3	52.3	300.6
Pt/$NaTaO_3$	10.7	54.7	295.1

a E_1 was calculated by the following equation: $E_1 = E_M + (E_1 - E_M) = 44.0$ kJ/mol + $(E_1 - E_M)$ with the evaporation enthalpy of water $E_M \approx 44$ kJ/mol and the obtained fitting parameter $E_1 - E_M$.

Supplementary Note 2
Supplementary data of photocatalytic reaction.

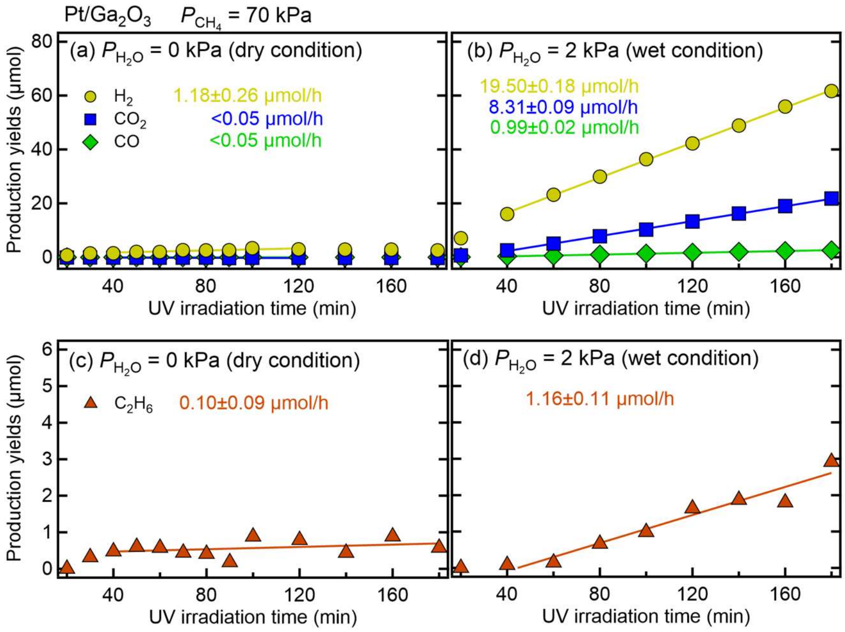

Figure S2-1. Impact of interfacial water on photocatalytic methane conversion rate. Typical time profiles of the production yields of H_2, CO_2, and CO produced on Pt/Ga_2O_3 photocatalysts as a function of UV irradiation time under P_{CH_4} = 70 kPa with the H_2O partial pressure (P_{H_2O}) of (a) 0 kPa and (b) 2 kPa, and those of C_2H_6 produced on Pt/Ga_2O_3 under (c) P_{H_2O} = 0 kPa and (d) P_{H_2O} = 2 kPa. Reaction products increased almost linearly with the UV irradiation time, implying that the photocatalytic reactions proceed under steady-state conditions. The linear fitting lines to evaluate the formation rates are also shown.

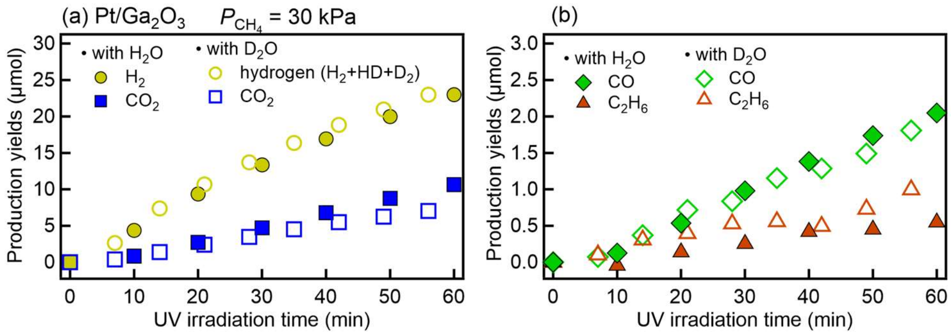

Figure S2-2. Demonstration of negligible water isotope effects on the photocatalytic methane conversion. Time profile of the production yields of (a) hydrogen (H_2, HD, and D_2), CO_2 and (b) CO, C_2H_6 produced on Pt/Ga_2O_3 photocatalysts under UV irradiation at the CH_4 pressure of 30 kPa and the water vapor (H_2O or D_2O) pressure of 2 kPa. Minimal isotope dependence indicates that kinetic isotope effect between H and D is negligible.

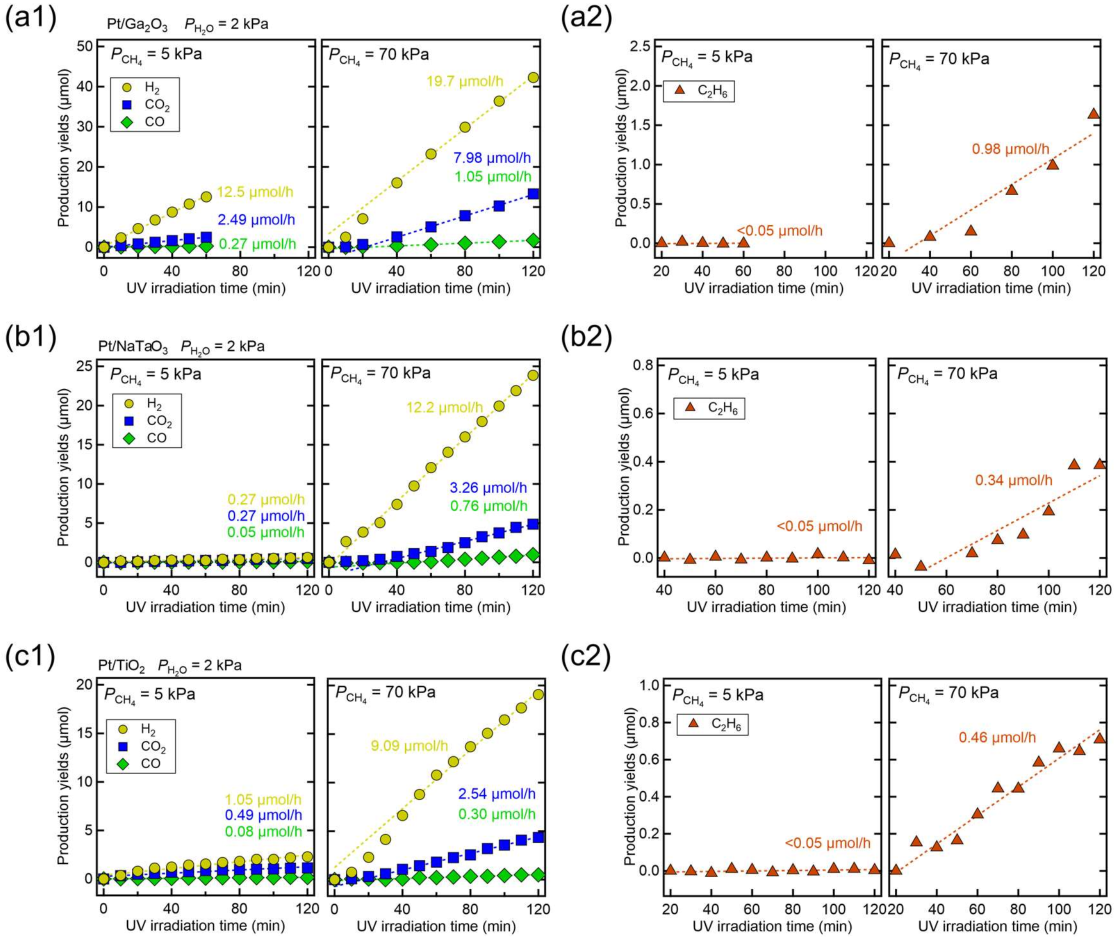

Figure S2-3. Production yields of H_2, CO_2, and CO using (a1) Pt/Ga_2O_3, (b1) Pt/$NaTaO_3$, and (c1) Pt/TiO_2 photocatalysts under UV irradiation for the CH_4 partial pressure of 5 kPa (left side) and 70 kPa (right side) at P_{H_2O} = 2 kPa. Production yields of C_2H_6 obtained using (a2) Pt/Ga_2O_3, (b2) Pt/$NaTaO_3$, and (c2) Pt/TiO_2 photocatalysts under the same reaction conditions. All photocatalyst samples showed higher yields for higher CH_4 partial pressure of 70 kPa. Reaction products increased almost linearly with increasing UV irradiation time, implying that the photocatalytic reactions proceed under steady-state conditions. The linear fitting lines to evaluate the formation rates are also shown.

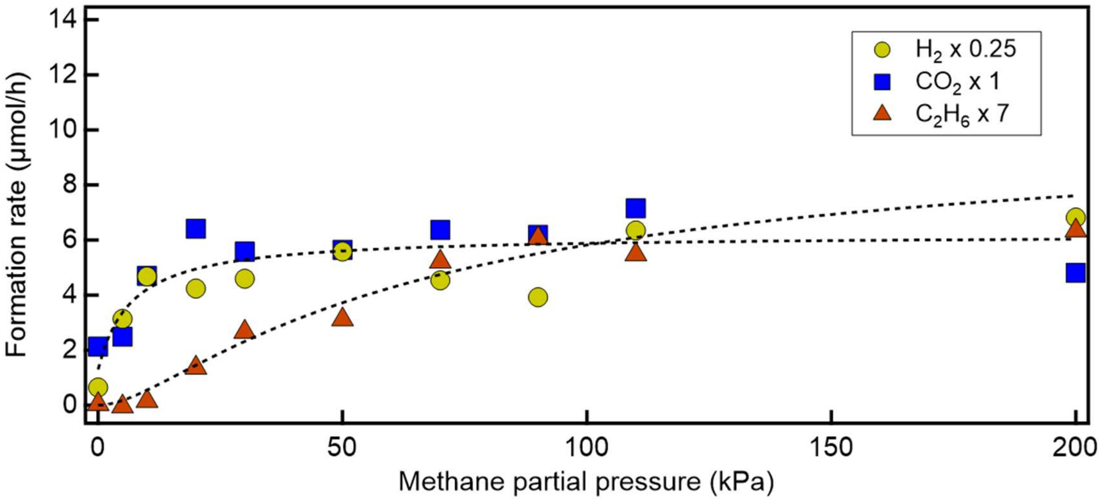

Figure S2-4. P_{CH_4} profiles of the formation rates of H_2, CO_2, and C_2H_6 using Pt/Ga_2O_3 photocatalysts under UV irradiation under wet conditions (P_{H_2O} = 2 kPa). The formation rates observed at the higher partial pressure of methane (P_{CH_4} = 200 kPa) are also shown.

Supplementary Note 3
IR spectra of HDO molecules on photocatalyst surfaces.

As shown in Fig. 2, we observed that the IR peaks in the O–H stretching region (3000–3600 cm^{-1}) increased in intensity during the photocatalytic methane (CH_4) conversion reaction using isotope-labeled D_2O water. The small O–H stretching band observed in the D_2O-dominant environment is mostly derived from adsorbed HDO molecules[4] that are produced by hydrogen abstraction of OD radicals from CH_4 (equation (1)).

Although the isotope-diluted O–H stretching band of HDO in D_2O typically shows a relatively sharp peak[5], the observed O–H stretching band under the specified reaction conditions had a broad structure (Fig. 2). This rather broad feature is mostly due to the inhomogeneous broadening derived from the inhomogeneous distribution of surface-adsorbed sites[6] as indicated by the spectral broadening of the intentionally adsorbed isotope-diluted HDO in the D_2O molecule (Fig. S3-1b).

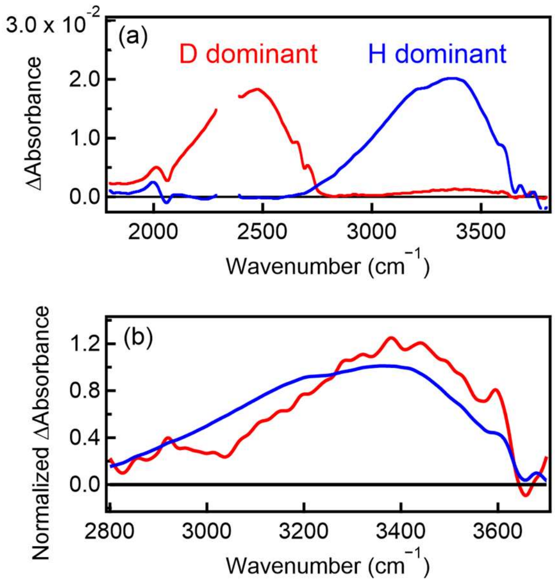

Figure S3-1. (a) IR spectra of water isotopes adsorbed on Pt/Ga_2O_3. The red spectrum was obtained when the surface was exposed to 4 Pa of D_2O vapor with a little HDO contaminant. Under this condition, presence of adsorbed H_2O is almost negligible under the equilibrium of H–D exchange[4]. The blue spectrum was obtained when the surface was exposed to 8 Pa of pure-H_2O vapor. (b) Peak-normalized IR spectra of O−H stretching band of pure H_2O and HDO diluted in D_2O on the Pt/Ga_2O_3 surfaces.

Supplementary Note 4
SEM and TEM images of the photocatalyst samples.

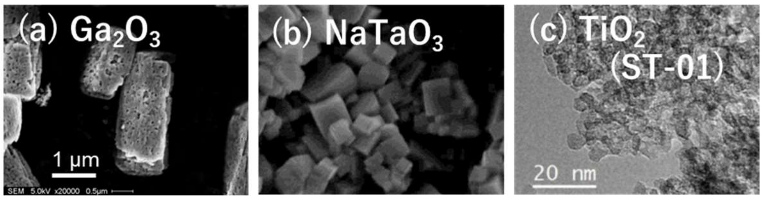

Figure S4-1. Scanning electron microscopy (SEM) images of (a) Ga_2O_3, (b) $NaTaO_3$ photocatalysts, and a tunneling electron microscopy (TEM) image of (c) TiO_2 (ST-01) photocatalysts. The typical particle size is ~3 μm (Ga_2O_3), ~200 nm ($NaTaO_3$), and ~3 nm (TiO_2), respectively.

Supplementary Note 5

MD simulation on the photocatalytic pre-activation of interfacial water under wet reaction condition.

The results from this study indicate that under dry conditions, methane is activated by direct hole transfer from the lattice O atom (O_{lat}) of the catalyst surface, as $CH_4 + h^+_{(O_{lat})} \rightarrow {}^{\bullet}CH_3 + H^+$. However, under wet conditions, methane is activated by $^{\bullet}OH$, which is generated from the interfacial water species ($CH_4 + {}^{\bullet}OH \rightarrow {}^{\bullet}CH_3 + H_2O$). To obtain an atomistic picture of these processes, we conducted *ab initio* molecular dynamics (AIMD) simulations of methane activation and preferential water activation ($H_2O + h^+_{(O_{lat})} \rightarrow {}^{\bullet}OH + H^+$). For methane activation under wet conditions, potential energy curve (PEC) snapshots of the MD trajectory are shown in the main text. Here, we present the supporting details of our MD simulations for the other processes.

5-1. Hole trapping on O atom of Ga_2O_3 surface.

First, we investigated the Ga_2O_3 surface with photogenerated holes. Our main focus was to identify the hole centers in Ga_2O_3. As stated in the Computational Details section of the main text, the hybrid-DFT method was applied for the hole-state calculation.

Fig. S5-1a shows the unit cell used in the calculations. The unit cell includes the β-Ga_2O_3, and 34 H_2O molecules, to model the Ga_2O_3 surface with one interfacial water layer. The (100) surface of Ga_2O_3 is exposed. Fig. S5-1b shows a snapshot of the MD trajectory for the mono-cationic Ga_2O_3 surface. The blue sphere shows an atom with a Mulliken spin population > 0.5 e. The sphere is localized in the surface O atom of the bridge site. During the MD calculation, the hole is mostly localized on this atom. From this result, we can conclude the photo-excitation leads surface localized holes on this O atom.

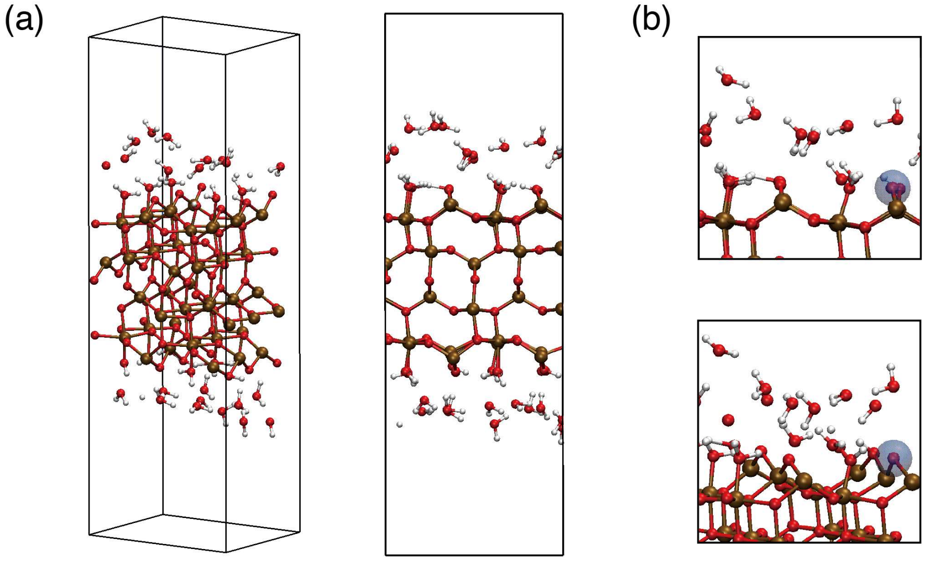

Figure S5-1. (a) The unit cell of the β-Ga_2O_3 (100) with the interfacial water layer. (b) Snapshot of the MD trajectory for the photo-excited β-Ga_2O_3. The blue sphere indicates the atom with a Mulliken spin population > 0.5 e. A view of the snapshot from another angle is also shown.

5-2. Photocatalytic activation of interfacial water.

The experimental results suggest that prior to CH_4 activation, $^{\bullet}OH$ is formed from the interfacial water and the hole on the Ga_2O_3 surface ($H_2O + h^{+}{}_{(O_{lat})} \rightarrow {}^{\bullet}OH + H^{+}$). We simulated this process using AIMD. Here, two bond lengths, Ga– O_{H_2O} and O_{lat}–H, were employed as the reaction coordinates, where O_{H_2O} denotes the O atom of the water molecule.

In Fig. S5-2, the PEC for $^{\bullet}OH$ formation and snapshots of the MD trajectory are shown. In the figure, the $^{\bullet}OH$ formation proceeds from left to right. The leftmost figure corresponds to the reactant state, where Ga– O_{H_2O} and O_{lat}–H are set at 2.0 and 1.0 Å, respectively. This corresponds to the $H_2O + h^{+}{}_{(O_{lat})}$ state, and we can observe that H_2O is adsorbed on the Ga atom, and the hole exists on O_{lat} as the blue sphere (Mulliken spin population > 0.5 e) is localized there. By shortening the O_{lat}–H distance, the H atom was abstracted from H_2O by O_{lat}. Along with this H-abstraction, hole transfer from O_{lat} to H_2O occurs, as seen from the transfer of the blue sphere from O_{lat} to H_2O. We can see that a hole exists in the $^{\bullet}OH$ part after H-abstraction, indicating $^{\bullet}OH$ formation from the interfacial water. The transfer of the proton and the hole (electron) occurs simultaneously, which is termed proton-coupled electron transfer (PCET). From the PEC, the reaction energy (ΔE) and activation energy (E_a) are calculated to be −14.9 kJ/mol and 21.2 kJ/mol, respectively.

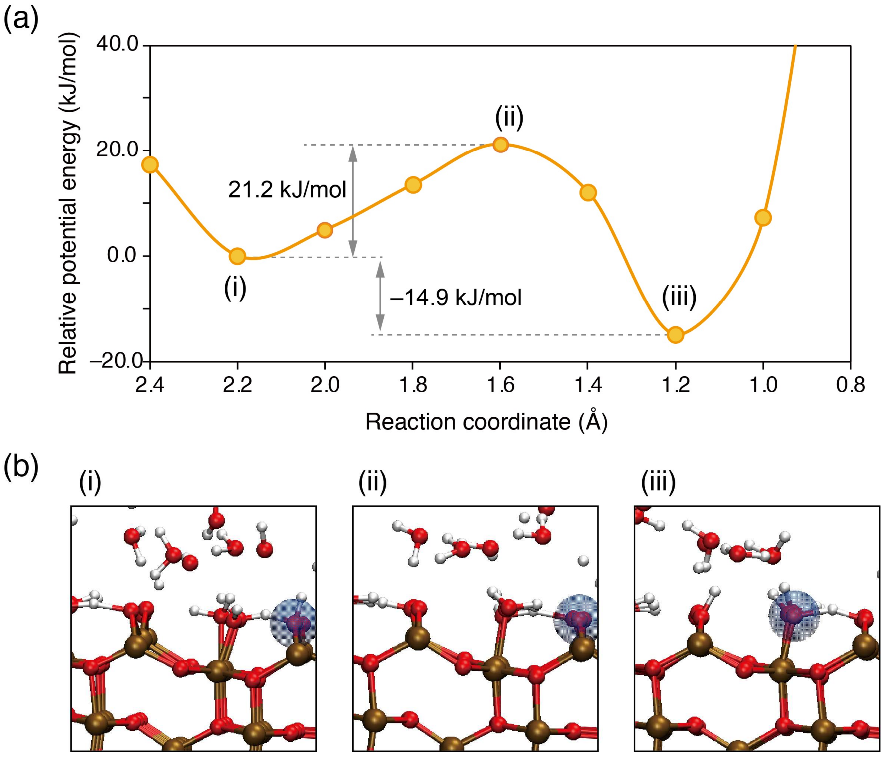

Figure S5-2. (a) Potential energy curve and (b) the snapshots for the •OH generation reaction from the hole-trapped surface O_{lat} atom and the H_2O molecule. The blue sphere indicates the atom with a Mulliken spin population > 0.5 e.

5-3. Computational results for the CH_4 activation in the dry condition.

We performed CH_4 activation by O_{lat}, in the absence of interfacial water molecules.

In Figs. S5-3a and S5-3b, the PEC of H-abstraction from CH_4 by O_{lat} is shown. As seen in the figure, H-abstraction proceeds without an activation barrier, and the reaction is exothermic ($\Delta E = -90.6$ kJ/mol). This result suggests that H-abstraction from CH_4 readily occurs under dry conditions.

In Fig. S5-3c, the optimized geometry for CH_3 adsorbed on O_{lat} is shown. The adsorption energy (E_{ads}) defined by $E_{ads} = E_{CH_3\text{-}Ga_2O_3} - (E_{CH_3} + E_{Ga_2O_3})$ is −171.9 kJ/mol, which suggests a strong adsorption of CH_3 on Ga_2O_3. Hence, desorption of CH_3 is unlikely to occur, and overoxidation on the surface to create other hydrocarbon species and coke[7-9] is likely to occur.

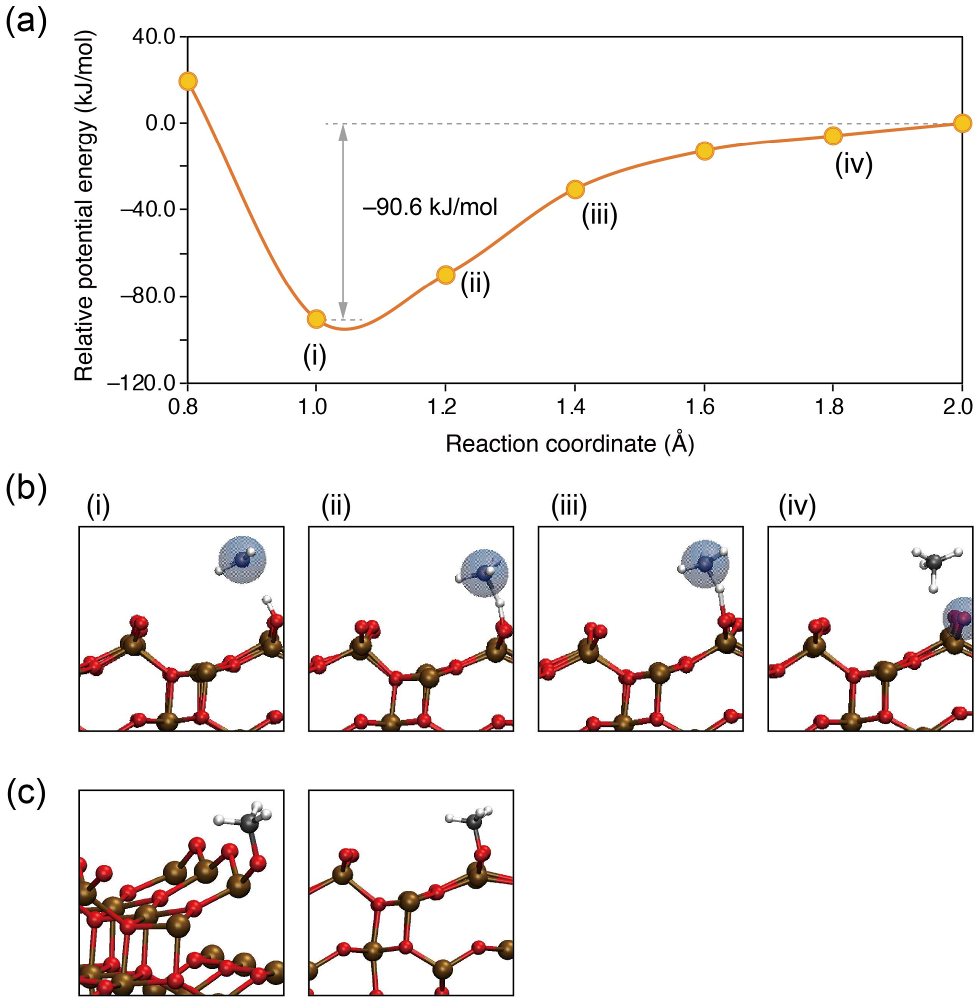

Figure S5-3. (a) The potential energy curve and (b) snapshot for CH_4 activation by the surface O_{lat} atom under dry conditions. The blue sphere indicates the atom with a Mulliken spin population > 0.5 e. (c) The optimized geometry for the CH_3 adsorbed on the Ga_2O_3 surface, under dry conditions. View images of the two angles are shown.

Supplementary Note 6
Kinetic analysis for derivation of methane conversion rates.

Under wet reaction conditions where photocatalyst surfaces are covered with ~1 ML of water molecules and the photoactivation of interfacial water is not rate-determining, the methane transformation process at the photocatalyst surface is divided into three steps, as shown in Figs. 5 and S6-1: (i) adsorption and desorption of methane at vacant active sites, (ii) sequential reactions of surface intermediate species, and (iii) desorption of the final surface products. In the first step, methane can undergo molecular physisorption ($X_1 = CH_4$) or dissociative chemisorption ($X_1 = {}^{\bullet}CH_3$), as shown in Figs. 5 and S6-1. Based on our *operando* IR absorption measurements (Fig. 2) and MD simulations (Fig. 3), methane molecules are likely to adsorb dissociatively as methyl radicals ($X_1 = {}^{\bullet}CH_3$) in photocatalytic methane conversion with water. Kinetic analysis, detailed in this note, further supports this scenario.

The methyl radical X_1 species reacts with another methyl radical to form C_2H_6 (Fig. S6-1a) or further oxidized to form the second intermediate surface species denoted as X_2 (CH_2 or CH_3OH species), as shown in Figs. S6-1b and S5-1c. The intermediate species X_2 is further converted to CO (X_6) or CO_2 (X_8) via multiple intermediate species at the photocatalyst surfaces (Figs. S6-1b and S5-1c). The surface-adsorbed product species, denoted as *m*-th intermediate (X_m: $m = 2$ for C_2H_6 (Fig. S6-1a), $m = 6$ for CO, and $m = 8$ for CO_2 (Fig. S6-1b)) desorbed as a gaseous product. In the following section 6-1 and 6-2, P_{CH_4} dependence of C_2H_6 and CO/CO_2 formation rates are described, respectively.

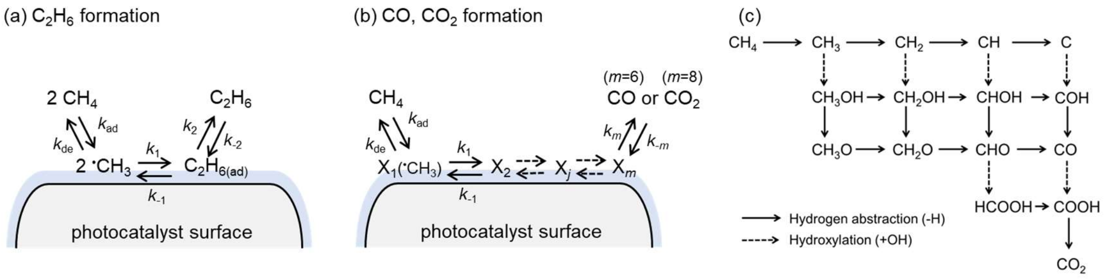

Figure S6-1. Schematic of kinetic models for (a) ethane formation, (b) carbon monoxide ($m = 6$) formation, and carbon dioxide ($m = 8$) formation on photocatalyst surfaces covered with ~1 ML of water molecules. The first intermediate X_1 corresponds to the ${}^{\bullet}CH_3$ in the photocatalytic methane conversion in water. (c) Diagram showing oxidation and possible reaction intermediate species from CH_4 to CO_2[10,11]. The number of intermediates *m* in CO and CO_2 formation processes are assumed to be six and eight, respectively on the basis of the number of holes that are needed to convert CH_4.

6-1. Reaction model of ethane formation process.

Operando spectroscopy measurements (Fig. 2) and MD simulations (Fig. 3) showed that hydrogen abstraction of methane by photoactivated interfacial water species (equation (1)) is the first step in the photocatalytic oxidation of methane using water.

$$CH_{4(gas)} + {}^{\bullet}OH_{(ad)}(*_{v}) \underset{k_{de}}{\overset{k_{ad}}{\rightleftarrows}} {}^{\bullet}CH_{3(ad)} + H_2O_{(ad)}\,. \qquad (S6\_1)$$

As shown in Fig. S6-1a, ethane gas was produced by the coupling reaction of the two adsorbed $^{\bullet}CH_{3(ad)}$ species, followed by the desorption of surface-adsorbed $C_2H_{6(ad)}$ species:

$$2\,{}^{\bullet}CH_{3(ad)} + *_{v'} \underset{k_{-1}}{\overset{k_1}{\rightleftarrows}} C_2H_{6(ad)} + 2\,*_{v}\,, \qquad (S6\_2)$$

$$C_2H_{6(ad)} \underset{k_{-2}}{\overset{k_2}{\rightleftarrows}} C_2H_{6(gas)} + *_{v'}\,. \qquad (S6\_3)$$

where $*_{v}$ and $*_{v'}$ denote vacant surface sites for methyl radicals and ethane, respectively. From the following kinetic analysis, P_{CH_4} dependence of ethane formation rate ($R_{C_2H_6}$) was derived as $R_{C_2H_6} \propto [K_0P_{CH_4}/(1 + K_0P_{CH_4})]^2$.

The net adsorption rate of methane R_0 during the initial methane activation process (equation (S6_1)) can be described as,

$$R_0 = k_{ad}P_{CH_4}N_v - k_{de}N_{CH_3} = k_{ad}P_{CH_4}N_{max}\left(\theta_v - K_0^{-1}P_{CH_4}^{-1}\theta_{CH_3}\right), \qquad (S6\_4)$$

where k_{ad} and k_{de} are the rate constants of methane adsorption and desorption, respectively, and K_0 denotes the equilibrium constants of adsorption and desorption ($K_0 \equiv k_{ad}/k_{de}$). The population of methyl radicals and the number of vacant sites for methyl radicals ($*_{v}$) are denoted as N_{CH_3} and N_v, respectively. Their coverages are denoted as θ_{CH_3} ($\equiv N_{CH_3}/N_{max}$) and θ_v ($\equiv N_v/N_{max}$), where N_{max} is the total number of dissociative adsorption sites for methane, i.e., $^{\bullet}$OH radical sites existing on the catalysts under working conditions. The rates of the surface reactions R_1 and R_2 (equations (S6_1) and (S6_2)) can be expressed as follows:

$$R_1 = k_1N_{CH_3}^2N_{v'} - k_{-1}N_{C_2H_6}N_v^2 = k_1N_{max}^2N_{max'}\left(\theta_{CH_3}^2\theta_{v'} - K_1^{-1}\theta_{C_2H_6}\theta_v^2\right), \qquad (S6\_5)$$

$$R_2 = k_2N_{C_2H_6} - k_{-2}P_{C_2H_6}N_{v'} = k_2N_{max'}\left(\theta_{C_2H_6} - K_2^{-1}P_{C_2H_6}\theta_{v'}\right), \qquad (S6\_6)$$

where k_1 and k_2 are the rate constants of the coupling reaction of the two adsorbed $^{\bullet}CH_{3(ad)}$ species (equation (S6_2)) and desorption of the adsorbed $C_2H_{6(ad)}$ species (equation (S6_3)), k_{-1} and k_{-2} are the rate constants of the backward reactions, and K_1 ($\equiv k_1/k_{-1}$) and K_2 ($\equiv k_2/k_{-2}$) denote the equilibrium constants of these reactions. The population of adsorbed ethane and the number of vacant sites for ethane ($*_{v'}$) are denoted as $N_{C_2H_6}$ and $N_{v'}$, respectively. Their coverages are denoted as $\theta_{C_2H_6}$ ($\equiv N_{C_2H_6}/N_{max'}$) and $\theta_{v'}$ ($\equiv N_{v'}/N_{max'}$), where $N_{max'}$ is the total number of adsorption sites for ethane. Because the amount of

produced gas increases linearly with time (Figs. S2-1 and S2-3) and the consumption of the inlet CH_4 gas (< 0.1 kPa) is negligible in our reaction experiments, it was assumed that these reactions occur at a steady rate as,

$$0.5R_0 = R_1 = R_2. \quad (S6\_7)$$

In case the coupling reaction (equation ($S6\_2$)) is rate-determining, $k_{ad}P_{CH_4}N_{max} \gg R_1$ and $k_2N_{max'} \gg R_1$ are satisfied. Based on these relations and equation ($S6\_7$) ($k_{ad}P_{CH_4}N_{max} \gg R_1 = 0.5R_0$ and $k_2N_{max'} \gg R_1 = R_2$), θ_{CH_3} and $\theta_{C_2H_6}$ are expressed by θ_v and $\theta_{v'}$ as follows:

$$0.5R_0/\left(k_{ad}P_{CH_4}N_{max}\right) = 0.5\left(\theta_v - K_0^{-1}P_{CH_4}^{-1}\theta_{CH_3}\right) \ll 1, \quad (S6\_8)$$

$$\theta_{CH_3} \approx K_0P_{CH_4}\theta_v. \quad (S6\_9)$$

$$R_2/(k_2N_{max'}) = \theta_{C_2H_6} - K_2^{-1}P_{C_2H_6}\theta_{v'} \ll 1, \quad (S6\_10)$$

$$\theta_{C_2H_6} \approx K_2^{-1}P_{C_2H_6}\theta_{v'}. \quad (S6\_11)$$

Because the numbers of total adsorption sites (sum of vacant and filled sites) for $^{\bullet}CH_3$ and C_2H_6 are constant ($N_{max} = N_v + N_{CH_3}$, $N_{max'} = N_{v'} + N_{C_2H_6} \Leftrightarrow 1 = \theta_v + \theta_{CH_3}$, $1 = \theta_{v'} + \theta_{C_2H_6}$), θ_v, θ_{CH_3}, $\theta_{v'}$, and $\theta_{C_2H_6}$ can be expressed as follows using equations ($S6\_9$) and ($S6\_11$):

$$1 = \theta_v + \theta_{CH_3} = \theta_v\left(1 + K_0P_{CH_4}\right), \quad (S6\_12)$$

$$\therefore\ \theta_v = \frac{1}{1 + K_0P_{CH_4}}. \quad (S6\_13)$$

$$\theta_{CH_3} = K_0P_{CH_4}\theta_v = \frac{K_0P_{CH_4}}{1 + K_0P_{CH_4}}. \quad (S6\_14)$$

$$1 = \theta_{v'} + \theta_{C_2H_6} = \theta_{v'}\left(1 + K_2^{-1}P_{C_2H_6}\right), \quad (S6\_15)$$

$$\therefore\ \theta_{v'} = \frac{1}{1 + K_2^{-1}P_{C_2H_6}}. \quad (S6\_16)$$

$$\theta_{C_2H_6} = K_2^{-1}P_{C_2H_6}\theta_{v'} = \frac{K_2^{-1}P_{C_2H_6}}{1 + K_2^{-1}P_{C_2H_6}}. \quad (S6\_17)$$

Equation ($S6\_14$) is the Langmuir adsorption isotherm equation, which is given as equation (3) in the main text. From equations ($S6\_5$) and ($S6\_7$), the P_{CH_4} profile of the ethane formation rate ($R_2 \equiv R_{C_2H_6}$) is derived as follows:

$$R_{C_2H_6} \equiv R_2 = R_1 = k_1N_{max}^2N_{max'}\left(\theta_{CH_3}^2\theta_{v'} - K_1^{-1}\theta_{C_2H_6}\theta_v^2\right)$$

$$= k_1N_{max}^2N_{max'}\left[\left(\frac{K_0P_{CH_4}}{1 + K_0P_{CH_4}}\right)^2 - \frac{K_2^{-1}P_{C_2H_6}}{1 + K_2^{-1}P_{C_2H_6}}\left(\frac{K_1^{-1} + \left(K_0P_{CH_4}\right)^2}{\left(1 + K_0P_{CH_4}\right)^2}\right)\right]. \quad (S6\_18)$$

Notably, the inlet pressure of methane gas is in the order of 5–100 kPa was much higher than the pressure of the produced ethane gas ($P_{CH_4} \gg P_{C_2H_6}$) in our experiments. Therefore, the second term in equation ($S6\_18$) consisting of $P_{C_2H_6}$ is negligible compared to the first term consisting of P_{CH_4}, which corresponds to the following approximation:

$\theta_{V'} \approx 1$ and $\theta_{C_2H_6} \ll 1$. Thus, the P_{CH_4} dependence of $R_{C_2H_6}$ is derived as the square of the Langmuir adsorption isotherm equation,

$$R_{C_2H_6} \approx k_1 N_{max}^2 N_{max'} \theta_{CH_3}^2 = k_1 N_{max}^2 N_{max'} \left(\frac{K_0 P_{CH_4}}{1 + K_0 P_{CH_4}}\right)^2. \quad (S6\_19)$$

This equation indicates that the saturation value on the P_{CH_4} profile of the ethane formation rate is proportional to a factor N_{max}^2. N_{max} is the total number of active sites of methane activation (equations ($S6\_1$)); thus, it corresponds to the population of photoactivated water ($^{\bullet}OH$) stationarily existing on catalyst surfaces.

Next, we discuss the details of the equilibrium constant K_0. The adsorption and desorption rate constants are given by:

$$k_{ad} = \frac{sA}{\sqrt{2\pi M k_B T_g}} \exp\left(-\frac{E_{ad}}{k_B T_s}\right), \quad (S6\_20)$$

$$k_{de} = \nu_0 \exp\left(-\frac{E_{de}}{k_B T_s}\right), \quad (S6\_21)$$

where M is the mass of a CH_4 molecule (2.66×10^{-26} kg), k_B is the Boltzmann constant (1.38×10^{-23} J/K), T_g is the temperature of gaseous CH_4 (~296 K), T_s is the temperature of the photocatalyst surface (~318 K), ν_0 is the frequency factor[12] (1.0×10^{13} s^{-1}), A is the area occupied by adsorbed methane (1.0×10^{-15} cm^2), E_{ad} is the activation energy of dissociative adsorption of CH_4, E_{de} is the activation energy of desorption of CH_4 and s is the sticking coefficient of CH_4. The equilibrium constant K_0 is,

$$K_0 = \frac{sA}{\nu_0\sqrt{2\pi M k_B T_g}} \exp\left(\frac{E_{de} - E_{ad}}{k_B T_s}\right) \equiv \frac{sA}{\nu_0\sqrt{2\pi M k_B T_g}} \exp\left(\frac{U}{k_B T_s}\right), \quad (S6\_22)$$

where U is the stabilization energy of the adsorbed methyl radical (Fig. S6-2).

From equations ($S6\_14$), ($S6\_19$), and ($S6\_22$), the P_{CH_4} profiles of θ_{CH_3} and $R_{C_2H_6}$ were determined by U as shown in (Fig. S6-3). From equation ($S6\_22$), when the value of U increases, the equilibrium constant K_0 increases. With an increase in K_0, θ_{CH_3} and $R_{C_2H_6}$ increase sharply at low P_{CH_4} as shown in Figs. S6-3a and S6-3b. The reciprocal equilibrium constant K_0^{-1} indicates the critical pressure around which θ_{CH_3} drastically increases. When P_{CH_4} reaches K_0^{-1}, θ_{CH_3} becomes 0.5. Notably, the P_{CH_4} profiles of $R_{C_2H_6}$ show a sigmoidal curve on the horizontal axis displayed on a linear scale (Fig. S6-3d) and the pressure at which the curve shows the inflection point is K_0^{-1}, where $R_{C_2H_6}$ becomes $0.25\ k_1 N_{max}^2\ N_{max'}$.

If the $^{\bullet}CH_3$ radical intermediate is released into the gas phase and the subsequent self-coupling reaction to afford ethane proceeds in the gas phase, the production rate of ethane is given as the first order of θ_{CH_3}: $R_{C_2H_6} \propto \theta_{CH_3} = K_0 P_{CH_4}/(1 + K_0 P_{CH_4})$ (see the following section 6-3 for details). As shown by the experimental results (Fig. S6-4a), the

rate of C_2H_6 formation increased sigmoidally with P_{CH_4}; almost no increase was observed under conditions of low methane pressure (P_{CH_4} < 10 kPa), whereas it successively increased in the range of 20–80 kPa and saturated at ~100 kPa (1 atm). The observed sigmoidal behavior on the linear pressure scale cannot be described by the first-order contribution of θ_{CH_3} (Fig. S6-3c); rather, it is well fitted the second-order contribution of θ_{CH_3} (Fig. S6-3d), indicating that ethane was produced at the photocatalyst surface rather than in the gas phase.

As shown in Figs. S6-4b–d, the P_{CH_4} profiles of the C_2H_6 formation rates on the three photocatalysts were all fitted with equation (*S*6_19) using the fitting parameter K_0 listed in Table S6-1. Based on equation (*S*6_22), the stabilization energy *U* is expressed as follows:

$$U = k_B T_s \ln\left(\frac{K_0 \nu_0 \sqrt{2\pi M k_B T_g}}{sA}\right). \qquad (S6\_23)$$

The value of *U* (~40 kJ/mol) is significantly higher than that of molecularly physisorbed methane (12 kJ/mol) (Supplementary Note 8). This is consistent with the results showing that methane dissociatively chemisorbs on photocatalyst surfaces under UV irradiation in wet conditions.

It is assumed that the initial sticking coefficient (*s*) is 0.1. If the value of *s* varies by one digit, the value of *U* changes by ~6 kJ/mol. The probability value *s* cannot exceed 1; thus, the lowest limit of *U* is 6 kJ/mol less than the values listed in Table S6-1. The lower limit values of *U* (26–30 kJ/mol) are significantly higher than the physisorption energy of methane (~12 kJ/mol). Therefore, the uncertainty of *s* has no effect on the assumption that dissociative adsorption of methane is the first step in photocatalytic methane oxidation with water.

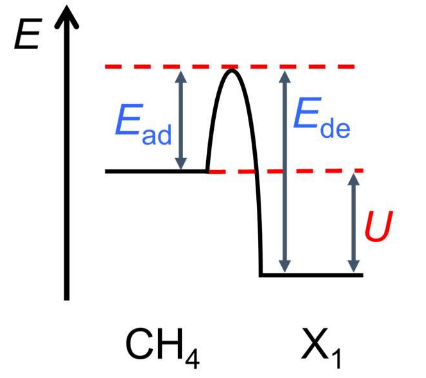

Figure S6-2. Schematic energy diagram of methane activation to X_1 (methyl radical).

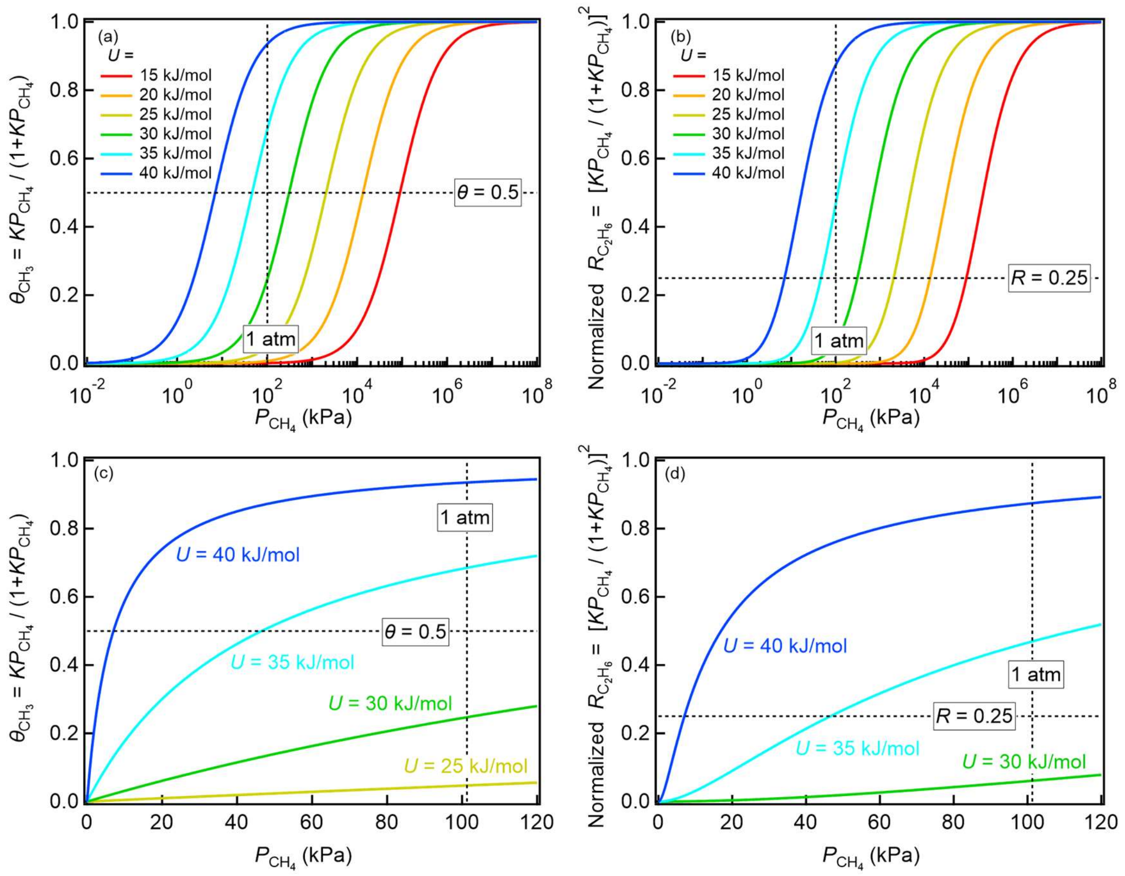

Figure S6-3. P_{CH_4} dependence of (a) θ_{CH_3} and (b) normalized $R_{C_2H_6}$ (= $\theta_{CH_3}{}^2$) at different values of U (15–40 kJ/mol) on the horizontal axis displayed in a logarithmic scale. U = 15, 20, 25, 30, 35, and 40 kJ/mol correspond to $K_0^{-1} = 9.0\times10^4$, 1.4×10^4, 2.1×10^3, 3.1×10^2, 4.7×10^1, and 7.1×10^0 kPa, respectively. P_{CH_4} dependence of (c) θ_{CH_3} and (d) normalized $R_{C_2H_6}$ (= $\theta_{CH_3}{}^2$) on the horizontal axis displayed in a linear scale. These curves were obtained with a typical sticking coefficient of CH_4 (s) of 0.1.

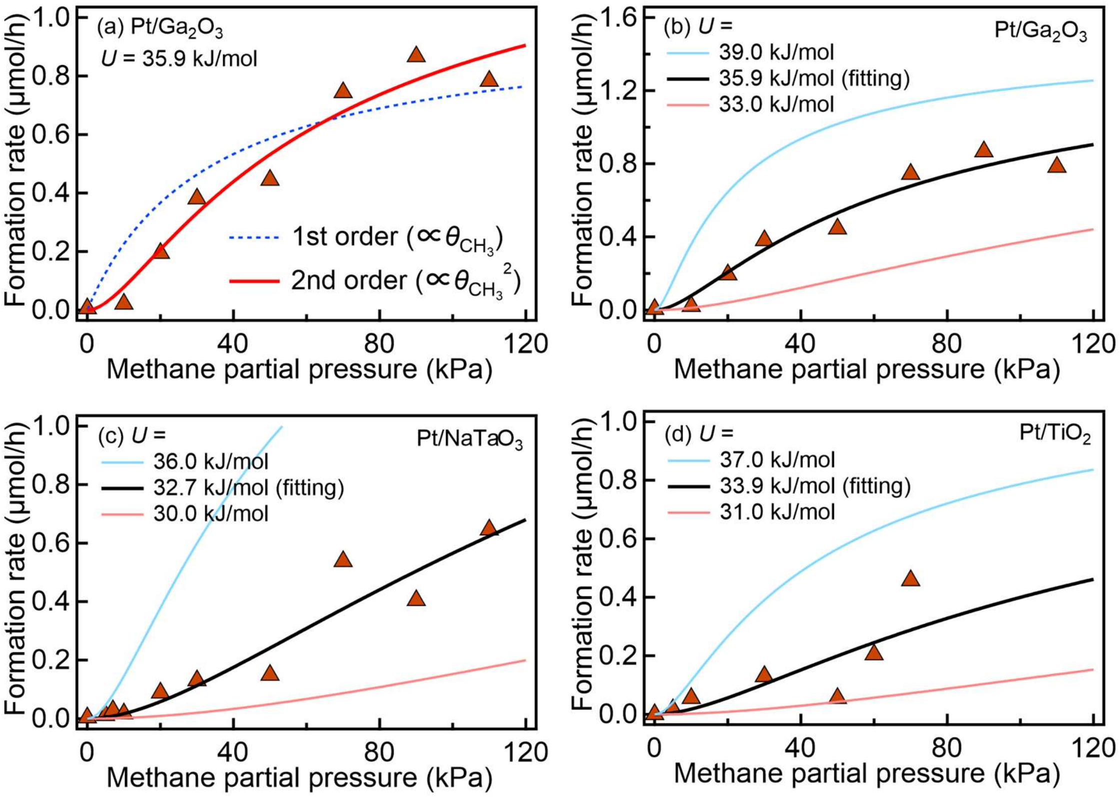

Figure S6-4. (a) Formation rates of C_2H_6 for Pt/Ga_2O_3 photocatalysts under UV irradiation as a function of partial pressure of methane at an H_2O partial pressure of 2 kPa. The red line results from curve fitting with the second order of Langmuir-type equation (equation (*S*6_19)). The simulated curves with the first order of Langmuir-type equation are also shown for comparison. The experimentally observed sigmoidal curve is better fitted by the second-order equation. Formation rates of C_2H_6 for (b) Pt/Ga_2O_3 (Fig. S6-4a), (c) $Pt/NaTaO_3$, and (d) Pt/TiO_2 photocatalysts under UV irradiation as a function of partial pressure of methane at an H_2O partial pressure of 2 kPa. The solid lines represent the P_{CH_4} profiles simulated by equation (*S*6_19) with typical U values and $s = 0.1$.

Table S6-1. Fitting parameter K_0 for the P_{CH_4} profiles of ethane formation rates on three photocatalysts (Figs. 4 and S6-4) and stabilization energy (U) of methyl radicals estimated by equation (*S*6_23) with $s = 0.1$.

	K_0 (Pa^{-1})	K_0^{-1} (kPa)	U (kJ/mol)
Pt/Ga_2O_3	3.0×10^{-5}	33.3	35.9
$Pt/NaTaO_3$	8.9×10^{-6}	112.8	32.7
Pt/TiO_2	1.4×10^{-5}	71.1	33.9

6-2. Reaction model of carbon monoxide and carbon dioxide formation processes.

A number of reaction intermediates are involved following the methyl radical (= X_1) formation, as shown in Figs. S6-1b and S6-1c. Multiple reactions involving intermediates take place on the photocatalyst surface and subsequent desorption of the final product molecules is modeled as follows:

$$\mathrm{CH}_{4(\mathrm{gas})} + *_1 \underset{k_{\mathrm{de}}}{\overset{k_{\mathrm{ad}}}{\rightleftarrows}} \mathrm{X}_1, \qquad (S6\_24_0)$$

$$\mathrm{X}_1 + *_2 \underset{k_{-1}}{\overset{k_1}{\rightleftarrows}} \mathrm{X}_2 + *_1, \qquad (S6\_24_1)$$

$$\mathrm{X}_2 + *_3 \underset{k_{-2}}{\overset{k_2}{\rightleftarrows}} \mathrm{X}_3 + *_2, \qquad (S6\_24_2)$$

$$\vdots$$

$$\mathrm{X}_j + *_{j+1} \underset{k_{-j}}{\overset{k_j}{\rightleftarrows}} \mathrm{X}_{j+1} + *_j, \qquad (S6\_24_j)$$

$$\vdots$$

$$\mathrm{X}_{m-1} + *_m \underset{k_{-(m-1)}}{\overset{k_{m-1}}{\rightleftarrows}} \mathrm{X}_m + *_{m-1}, \qquad (S6\_24_{m-1})$$

$$\mathrm{X}_m \underset{k_{-m}}{\overset{k_m}{\rightleftarrows}} \mathrm{X}_{m(\mathrm{gas})} + *_m, \qquad (S6\_24_m)$$

where the intermediate species (j) is labeled as X_j (j = 1 for methyl radical), the final product molecule is labeled as X_m (m = 6 for CO and m = 8 for CO_2), and $*_j$ denotes the vacant surface sites for the X_j intermediates. The net adsorption rate of methane in the initial methane activation process (R_0), net reaction rate of j-th intermediate species (R_j), and net desorption rate of the final product molecule (R_m) are described as follows:

$$R_0 = k_{\mathrm{ad}} P_{\mathrm{CH}_4} N_{\mathrm{v}_1} - k_{\mathrm{de}} N_{\mathrm{X}_1} = k_{\mathrm{ad}} P_{\mathrm{CH}_4} N_{\mathrm{max}_1} \left(\theta_{\mathrm{v}_1} - K_0^{-1} P_{\mathrm{CH}_4}^{-1} \theta_{\mathrm{X}_1}\right), \qquad (S6\_25)$$

$$R_j = k_j N_{\mathrm{X}_j} N_{\mathrm{v}_{j+1}} - k_{-j} N_{\mathrm{X}_{j+1}} N_{\mathrm{v}_j} = k_j N_{\mathrm{max}_j} N_{\mathrm{max}_{j+1}} \left(\theta_{\mathrm{X}_j} \theta_{\mathrm{v}_{j+1}} - K_j^{-1} \theta_{\mathrm{X}_{j+1}} \theta_{\mathrm{v}_j}\right), \qquad (S6\_26)$$

$$R_m = k_m N_{\mathrm{X}_m} - k_{-m} P_{\mathrm{X}_m} N_{\mathrm{v}_m} = k_m N_{\mathrm{max}_m} \left(\theta_{\mathrm{X}_m} - K_m^{-1} P_{\mathrm{X}_m} \theta_{\mathrm{v}_m}\right), \qquad (S6\_27)$$

where K_j denotes the equilibrium constant of the j-th reaction ($K_j \equiv k_j/k_{-j}$), N_{X_j} and N_{v_j} are the number of filled and vacant surface sites for j-th intermediate species (X_j), and θ_{X_j} ($\equiv N_{X_j}/N_{max_j}$) and θ_{v_j} ($\equiv N_{v_j}/N_{max_j}$) are the coverage and the ratio of vacant surface sites for X_j, respectively. N_{max_j} is the sum of the number of filled and vacant surface sites for X_j.

The amount of gas produced increased linearly with time (Figs. S2-1 and S2-3), which indicates that these reactions take place at a steady rate, as follows:

$$R_0 = R_1 = R_2 = \cdots = R_m. \qquad (S6\_28)$$

In case the reaction of the i-th intermediate species is the rate-determining step, the relations $k_{ad}P_{CH_4}N_{max_1} >> R_i$ and $k_m N_{max_m} >> R_i$ are satisfied. Because of the relationships $k_{ad}P_{CH_4}N_{max_1} >> R_i = R_0$ and $k_m N_{max_m} >> R_i = R_m$, θ_{X_1} and θ_{X_m} are expressed by θ_{v_1} and θ_{v_m} as follows:

$$R_0/\left(k_{ad}P_{CH_4}N_{max_1}\right) = \theta_{v_1} - K_0^{-1}P_{CH_4}^{-1}\theta_{X_1} \ll 1, \quad (S6\_29)$$

$$\theta_{X_1} \approx K_0 P_{CH_4}\theta_{v_1}. \quad (S6\_30)$$

$$R_m/\left(k_m N_{max_m}\right) = \theta_{X_m} - K_m^{-1}P_{X_m}\theta_{v_m} \ll 1, \quad (S6\_31)$$

$$\theta_{X_m} \approx K_m^{-1}P_{X_m}\theta_{v_m}. \quad (S6\_32)$$

Assuming that the total number of surface sites (sum of vacant and filled sites) of X_1 and X_m species is constant,

$$1 = \theta_{v_1} + \theta_{X_1}, \quad (S6\_33)$$

$$\theta_{v_1} = \frac{1}{1 + K_0 P_{CH_4}}, \quad (S6\_34)$$

$$\theta_{X_1} = \frac{K_0 P_{CH_4}}{1 + K_0 P_{CH_4}}. \quad (S6\_35)$$

$$1 = \theta_{v_m} + \theta_{X_m}, \quad (S6\_36)$$

$$\theta_{v_m} = \frac{1}{1 + K_m^{-1}P_{X_m}}, \quad (S6\_37)$$

$$\theta_{X_m} = \frac{K_m^{-1}P_{X_m}}{1 + K_m^{-1}P_{X_m}}. \quad (S6\_38)$$

In particular, when the forward reaction of the $^{\bullet}CH_3$ (= X_1) species (equation ($S6\_24_1$)) is rate-determining, the relation $k_j N_{max_j} N_{max_{j+1}} >> R_1$ ($2 \leq j \leq m-1$) is satisfied. The following relationship between the j-th and (j+1)-th species is obtained from $k_j N_{max_j} N_{max_{j+1}} >> R_1 = R_j$:

$$R_j/\left(k_j N_{max_j} N_{max_{j+1}}\right) = \theta_{X_j}\theta_{v_{j+1}} - K_j^{-1}\theta_{X_{j+1}}\theta_{v_j} \ll 1, \quad (2 \leq j \leq m-1) \quad (S6\_39)$$

$$\theta_{X_j}\theta_{v_{j+1}} \approx K_j^{-1}\theta_{X_{j+1}}\theta_{v_j}. \quad (S6\_40)$$

$$\therefore \theta_{X_j}/\theta_{v_j} = \left(\theta_{X_{j+1}}/\theta_{v_{j+1}}\right)K_j^{-1}. \quad (S6\_41)$$

From the recurrence formula, $\theta_{X_j}/\theta_{v_j}$ is expressed with $\theta_{X_m}/\theta_{v_m}$ as

$$\theta_{X_j}/\theta_{v_j} = \left(\theta_{X_m}/\theta_{v_m}\right)\prod_{f=j}^{m-1} K_f^{-1}. \quad (S6\_42)$$

Using equation ($S6\_32$) ($\theta_{X_m}/\theta_{v_m} \approx K_m^{-1}P_{X_m}$),

$$\theta_{X_j}/\theta_{v_j} = K_m^{-1}P_{X_m}\prod_{f=j}^{m-1} K_f^{-1} = P_{X_m}\prod_{f=j}^{m} K_f^{-1}. \quad (S6\_43)$$

Assuming that the total number of surface sites of each intermediate species is constant,

$$1 = \theta_{\mathrm{v}_j} + \theta_{\mathrm{X}_j}, \qquad (S6\_44)$$

the following formula of θ_{X_j} is derived from equations (S6_43) and (S6_44).

$$(1 - \theta_{\mathrm{v}_j})/\theta_{\mathrm{v}_j} = P_{\mathrm{X}_m} \prod_{f=j}^{m} K_f^{-1}, \qquad (S6\_45)$$

$$\theta_{\mathrm{v}_j} = \frac{1}{1 + P_{\mathrm{X}_m} \prod_{f=j}^{m} K_f^{-1}}, \qquad (S6\_46)$$

$$\theta_{\mathrm{X}_j} = \frac{P_{\mathrm{X}_m} \prod_{f=j}^{m} K_f^{-1}}{1 + P_{\mathrm{X}_m} \prod_{f=j}^{m} K_f^{-1}}. \qquad (S6\_47)$$

Based on these relations and $R_{\mathrm{m}} = R_1 = k_1 N_{\mathrm{max}_1} N_{\mathrm{max}_2}\ (\theta_{\mathrm{X}_1}\theta_{\mathrm{v}_2} - K_1^{-1}\theta_{\mathrm{X}_2}\theta_{\mathrm{v}_1})$, $P_{\mathrm{CH_4}}$ dependence of R_m is derived as follows:

$$R_m = k_1 N_{\mathrm{max}_1} N_{\mathrm{max}_2} \left[\frac{K_0 P_{\mathrm{CH_4}}}{1 + K_0 P_{\mathrm{CH_4}}} - \frac{P_{\mathrm{X}_m} \prod_{f=2}^{m} K_f^{-1}}{1 + P_{\mathrm{X}_m} \prod_{f=2}^{m} K_f^{-1}} \left(\frac{K_1^{-1} + K_0 P_{\mathrm{CH_4}}}{1 + K_0 P_{\mathrm{CH_4}}}\right)\right]. \qquad (S6\_48)$$

Under the experimental conditions, the inlet pressure of the methane gas was much higher than the pressure of the produced gas ($P_{\mathrm{CH_4}} >> P_{\mathrm{X}_m}$). Therefore, the second term in equation (S6_48) consisting of P_{X_m} is negligible compared to the first term consisting of $P_{\mathrm{CH_4}}$, which corresponds to the following approximation: $\theta_{\mathrm{v}_j} \approx 1$ and $\theta_{\mathrm{X}_j} << 1$ ($2 \le j \le m-1$). Thus, the $P_{\mathrm{CH_4}}$ dependence of R_m is given by a simple equation similar to the Langmuir adsorption isotherm equation,

$$R_m \approx k_1 N_{\mathrm{max}_1} N_{\mathrm{max}_2} \theta_{\mathrm{X}_1} = k_1 N_{\mathrm{max}_1} N_{\mathrm{max}_2} \left(\frac{K_0 P_{\mathrm{CH_4}}}{1 + K_0 P_{\mathrm{CH_4}}}\right). \qquad (S6\_49)$$

This equation indicates that the saturation values on the $P_{\mathrm{CH_4}}$ profiles of the CO/CO_2 formation rate are proportional to a factor $N_{\mathrm{max}_1} N_{\mathrm{max}_2}$. N_{max_1} is the total number of dissociative adsorption sites for methane, i.e., $^{\bullet}$OH radical sites, and N_{max_2} is the total number of active reaction sites for $^{\bullet}CH_3$ radicals. The relationship between K_0 and the stabilization energy of the rate-determining intermediate U is given in equations (S6_22) and (S6_23).

As shown in Figs. S6-5b–d, the P_{CH4} profiles of CO and CO_2 formation rates on the three photocatalysts were all well fitted by equation (S6_49), with the fitting parameters K_0 listed in Table S6-2. The stabilization energy U was calculated using equation (S6_23) with $s = 0.1$ and is listed in Table S6-2. Note that the $P_{\mathrm{CH_4}}$ profiles of H_2 formation rates are also well fitted with the Langmuir-type equation because CO_2 formation ($CH_4 + 2H_2O \rightarrow CO_2 + 4H_2$) was the major reaction and $R_{\mathrm{H_2}} \approx 4R_{\mathrm{CO_2}}$ condition was satisfied.

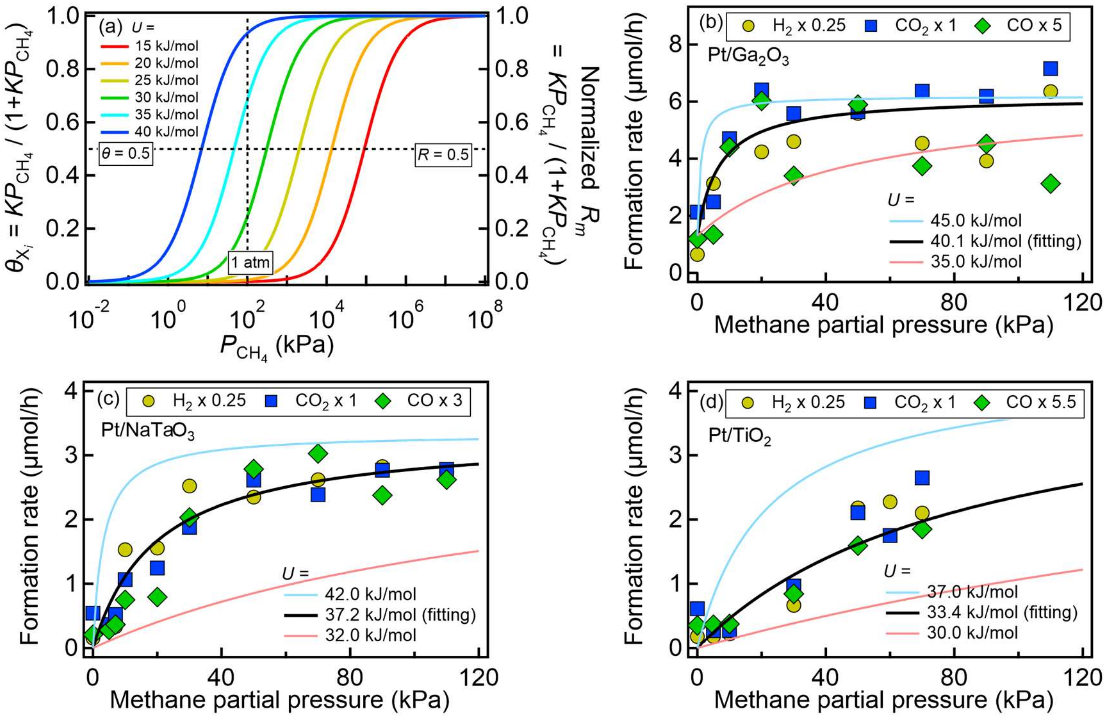

Figure S6-5. (a) P_{CH_4} (log scale) dependence of θ_{X_i} and normalized R_m (= θ_{X_i}) at various values of U (15–40 kJ/mol). Formation rates of CO_2, CO, and H_2 for (b) Pt/Ga_2O_3, (c) Pt/$NaTaO_3$, and (d) Pt/TiO_2 photocatalysts under UV irradiation as a function of partial pressure of methane at a H_2O partial pressure of 2 kPa. Solid lines represent the P_{CH_4} profiles simulated by the equation (equation (*S*6_23)) with typical values of U and s = 0.1.

Table S6-2. Fitting parameter K for the P_{CH_4} profiles of CO_2, CO, and H_2 formation rates in Figs. 4 and S6-6 on three different photocatalysts and calculated energy value U estimated by equation (*S*6_23) with s = 0.1.

	K (Pa^{-1})	K^{-1} (kPa)	U (kJ/mol)
Pt/Ga_2O_3	1.46×10^{-4}	6.8	40.1
Pt/$NaTaO_3$	5.00×10^{-5}	20.0	37.2
Pt/TiO_2	1.17×10^{-5}	85.2	33.4

It is important to discuss the case where the surface reaction of X_1 (equation ($S6\_24_1$)) is not the rate-determining step for the formation of CO and/or CO_2 because multiple reaction processes are mediated by various intermediates (Fig. S6-1c). However, almost the same P_{CH_4} profile for the formation rates of CO and CO_2 (equation ($S6\_49$)) is expressed as follows:

When the forward reaction of the i-th intermediate species ($X_i \leftrightarrow X_{i+1}$) is rate-determining, the relation $k_j >> R_i$ ($1 \leq j \leq m-1, j \neq i$) is satisfied. The following relationship between the j-th and (j+1)-th is obtained from $k_j >> R_i = R_j$.

$$R_j/\left(k_j N_{\max_j} N_{\max_{j+1}}\right) = \theta_{X_j}\theta_{v_{j+1}} - K_j^{-1}\theta_{X_{j+1}}\theta_{v_j} \ll 1, (1 \leq j \leq m-1, j \neq i). \quad (S6\_50)$$

$$\theta_{X_j}\theta_{v_{j+1}} \approx K_j^{-1}\theta_{X_{j+1}}\theta_{v_j}. \quad (1 \leq j \leq m-1, j \neq i). \quad (S6\_51)$$

If j is larger than i ($j > i$),

$$\theta_{X_j}/\theta_{v_j} = \left(\theta_{X_{j+1}}/\theta_{v_{j+1}}\right)K_j^{-1} = \left(\theta_{X_m}/\theta_{v_m}\right)\prod_{f=j}^{m-1} K_f^{-1} = P_{X_m}\prod_{f=j}^{m} K_f^{-1}. \quad (S6\_52)$$

If j is smaller than i ($j \leq i$),

$$\theta_{X_j}/\theta_{v_j} = \left(\theta_{X_{j-1}}/\theta_{v_{j-1}}\right)K_{j-1} = \left(\theta_{X_1}/\theta_{v_1}\right)\prod_{f=1}^{j-1} K_f = P_{CH_4}\prod_{f=0}^{j-1} K_f. \quad (S6\_53)$$

Assuming that the total number of surface sites of each intermediate species is constant,

$$1 = \theta_{v_j} + \theta_{X_j}, \quad (S6\_54)$$

The following formula for θ_{X_j} is derived from equations ($S6\_52$), ($S6\_53$), and ($S6\_54$).

$$(1-\theta_{v_j})/\theta_{v_j} = P_{X_m}\prod_{f=j}^{m} K_f^{-1}, \qquad (j > i) \quad (S6\_55)$$

$$\theta_{v_j} = \frac{1}{1 + P_{X_m}\prod_{f=j}^{m} K_f^{-1}}, \qquad (j > i) \quad (S6\_56)$$

$$\theta_{X_j} = \frac{P_{X_m}\prod_{f=j}^{m} K_f^{-1}}{1 + P_{X_m}\prod_{f=j}^{m} K_f^{-1}}. \quad (j > i) \quad (S6\_57)$$

$$(1-\theta_{v_j})/\theta_{v_j} = P_{CH_4}\prod_{f=0}^{j-1} K_f, \qquad (j \leq i) \quad (S6\_58)$$

$$\theta_{v_j} = \frac{1}{1 + P_{CH_4}\prod_{f=0}^{j-1} K_f}, \qquad (j \leq i) \quad (S6\_59)$$

$$\theta_{X_j} = \frac{P_{CH_4}\prod_{f=0}^{j-1} K_f}{1 + P_{CH_4}\prod_{f=0}^{j-1} K_f}. \quad (j \leq i) \quad (S6\_60)$$

Therefore, the P_{CH_4} profile of R_m is derived as follows:

$$R_m = R_i = k_i N_{\max_i} N_{\max_{i+1}}\left(\theta_{X_i}\theta_{v_{i+1}} - K_i^{-1}\theta_{X_{i+1}}\theta_{v_i}\right), \quad (S6\_61)$$

$$R_m = k_i N_{\max_i} N_{\max_{i+1}} \left[\frac{K' P_{CH_4}}{1 + K' P_{CH_4}} - \frac{P_{X_m} \prod_{f=i+1}^{m} K_f^{-1}}{1 + P_{X_m} \prod_{f=i+1}^{m} K_f^{-1}} \left(\frac{K_i^{-1} + K' P_{CH_4}}{1 + K' P_{CH_4}} \right) \right], \quad (S6\_62)$$

where K' is defined as $K' \equiv \prod_{f=0}^{i-1} K_f = K_0 K_1 K_2 \cdots K_{i-1}$. Assuming that P_{X_m} in equation (*S*6_62) is negligible compared with P_{CH_4}, the P_{CH_4} dependence of R_m is given by a simple equation similar to the Langmuir adsorption isotherm,

$$R_m \approx k_i N_{\max_i} N_{\max_{i+1}} \theta_{X_i} = k_i N_{\max_i} N_{\max_{i+1}} \left(\frac{K' P_{CH_4}}{1 + K' P_{CH_4}} \right). \quad (S6\_63)$$

Here, we discuss physicochemical implications of the effective constant K'. The equilibrium constant K_j is defined as follows:

$$K_j \equiv \frac{k_j}{k_{-j}} = \exp\left(-\frac{G_{j+1} - G_j}{k_B T_s} \right) \quad (1 \le j \le m-1), \quad (S6\_64)$$

where G_j is the free energy of the j-th intermediate, X_j (Fig. S6-6). Therefore, the effective constant K' can be expressed as follows:

$$\begin{aligned} K' &\equiv \prod_{f=0}^{i-1} K_f = K_0 K_1 K_2 \cdots K_{i-1} \\ &= \frac{sA}{\nu_0 \sqrt{2\pi M k_B T_g}} \exp\left(\frac{U}{k_B T_s} \right) \exp\left(-\frac{G_2 - G_1}{k_B T_s} \right) \exp\left(-\frac{G_3 - G_2}{k_B T_s} \right) \cdots \exp\left(-\frac{G_i - G_{i-1}}{k_B T_s} \right) \\ &= \frac{sA}{\nu_0 \sqrt{2\pi M k_B T_g}} \exp\left(\frac{U + G_1 - G_i}{k_B T_s} \right) \equiv \frac{sA}{\nu_0 \sqrt{2\pi M k_B T_g}} \exp\left(\frac{U'}{k_B T_s} \right), \quad (S6\_65) \end{aligned}$$

$$U' = k_B T_s \ln \left(\frac{K' \nu_0 \sqrt{2\pi M k_B T_g}}{sA} \right), \quad (S6\_66)$$

where $U' \equiv U + G_1 - G_i$ is the stabilization energy of the rate-determining i-th intermediate (Fig. S6-6). These are the same equations as those in the case where the forward reaction step of $^{\bullet}CH_3$ species ($X_1 \leftrightarrow X_2$) is the rate-determining step (equations (*S*6_22) and (*S*6_23)). These findings indicate that, regardless of which of the surface reactions is rate-determining, P_{CH_4} dependence of CO and CO_2 formation rates can be expressed by the same functional form as the Langmuir adsorption isotherm equation (equations (*S*6_49) and (*S*6_63)), and we can estimate U' from parameter K'. In the main text and Fig. S6-5 and Table S6-2 caption, K_0, U and K', U' were not distinguished and were represented as K and U for simplicity.

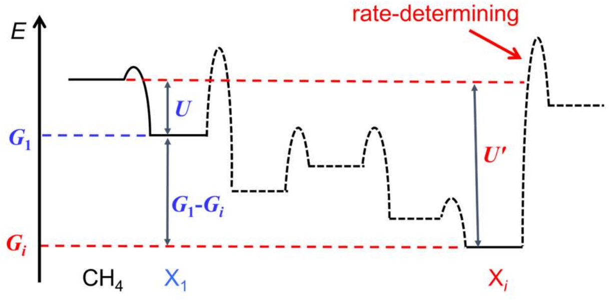

Figure S6-6. Schematic energy diagram of the methane oxidation process toward the rate-determining X_i species.

6-3. Possibility of the methyl radical coupling to ethane in gas phase.

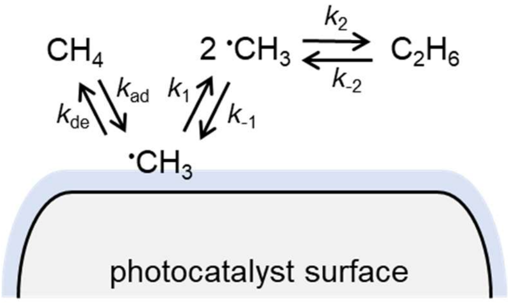

Figure S6-7. Schematic of the kinetic model for ethane formation derived from the coupling reaction of gaseous methyl radicals.

In the case of thermo-catalytic oxidative coupling of methane ($CH_4 + O_2 \rightarrow C_2H_6$, C_2H_4), C_2 species are supposedly produced by the coupling reaction of methyl radicals released in the gas phase[13]. In this section, we first derive the P_{CH_4} dependence of the ethane formation rate in the case where ethane is produced by the homocoupling reaction of methyl radicals in the gas phase (Fig. S6-7) and then discuss that the possibility of a gas-phase reaction is rejected in our experiments.

The reactions shown in Fig. S6-7 are modeled as follows,

$$\mathrm{CH}_{4(\mathrm{gas})} + *_\mathrm{v} \underset{k_\mathrm{de}}{\overset{k_\mathrm{ad}}{\rightleftarrows}} {}^\bullet\mathrm{CH}_{3(\mathrm{ad})}\,, \qquad (S6\_67)$$

$${}^\bullet\mathrm{CH}_{3(\mathrm{ad})} \underset{k_{-1}}{\overset{k_1}{\rightleftarrows}} {}^\bullet\mathrm{CH}_{3(\mathrm{gas})} + *_\mathrm{v}\,, \qquad (S6\_68)$$

$$2\,{}^\bullet\mathrm{CH}_{3(\mathrm{gas})} \underset{k_{-2}}{\overset{k_2}{\rightleftarrows}} \mathrm{C_2H}_{6(\mathrm{gas})}\,, \qquad (S6\_69)$$

$R_0 = k_{ad}P_{CH_4}N_{max}(\theta_v - K_0^{-1}\theta_{CH_3})$, $R_1 = k_1N_{max}(\theta_{CH_3} - K_1^{-1}P_{CH_3(gas)}\theta_v)$, $R_2 = k_2(P_{CH_3(gas)}{}^2 - K_2^{-1}P_{C_2H_6})$. Notably, the forward reaction of the surface-adsorbed methyl radicals (equation ($S6\_67$)) is first-order desorption of the gas phase. Because the amount of gas produced increases linearly with time (Figs. S2-1 and S2-3) and the consumption of inlet CH_4 (< 0.1 kPa) is negligible in our study, it was assumed that these reactions take place in steady state:

$$R_0 = R_1 = 2R_2. \qquad (S6\_70)$$

When desorption of the methyl radical (equation ($S6\_68$)) is rate-determining, the relationship $k_{ad}P_{CH_4}N_{max} \gg R_1 = R_0$ is satisfied, θ_{CH_3} is expressed by θ_v as follows:

$$R_0/(k_{ad}P_{CH_4}N_{max}) = \theta_v - K_0^{-1}P_{CH_4}^{-1}\theta_{CH_3} \ll 1, \qquad (S6\_71)$$

$$\theta_{CH_3} \approx K_0P_{CH_4}\theta_v. \qquad (S6\_72)$$

As the total number of surface sites is constant ($N_{max} = N_v + N_{CH_3} \Leftrightarrow 1 = \theta_v + \theta_{CH_3}$), θ_v and θ_{CH_3} can be expressed as follows:

$$1 = \theta_v + \theta_{CH_3} \approx \theta_v(1 + K_0P_{CH_4}), \qquad (S6\_73)$$

$$\therefore\ \theta_{\mathrm{v}} = \frac{1}{1 + K_0 P_{\mathrm{CH_4}}}. \qquad (S6\_74)$$

$$\theta_{\mathrm{CH_3}} = K_0 P_{\mathrm{CH_4}} \theta_{\mathrm{v}} = \frac{K_0 P_{\mathrm{CH_4}}}{1 + K_0 P_{\mathrm{CH_4}}}. \qquad (S6\_75)$$

Equation (*S*6_75) is the Langmuir adsorption isotherm equation. Assuming that $P_{\mathrm{CH_3(gas)}}$ is negligible compared to $P_{\mathrm{CH_4}}$, the $P_{\mathrm{CH_4}}$ profile of the ethane formation rate is described by the first-order Langmuir adsorption isotherm equation,

$$R_{\mathrm{C_2H_6}} \equiv R_2 = 0.5R_1 \approx 0.5k_1 N_{\mathrm{max}} \theta_{\mathrm{CH_3}} = 0.5k_1 N_{\mathrm{max}} \left(\frac{K_0 P_{\mathrm{CH_4}}}{1 + K_0 P_{\mathrm{CH_4}}} \right). \qquad (S6\_76)$$

The experimentally obtained ethane formation rates (Fig. S6-4a) shows the $P_{\mathrm{CH_4}}$ profiles expressed by the square of the Langmuir adsorption isotherm (equation (*S*6_19)). This result indicates that the methane coupling reaction occurs on the photocatalyst surface (Fig. S6-1), but not in the gas phase for all photocatalyst samples.

Supplementary Note 7
IR absorption spectroscopy in the C–H stretching region.

DRIFT spectra were recorded under steady-state reaction conditions at various partial pressures of methane (P_{CH_4}) on the Pt/Ga_2O_3 photocatalyst. In the C–H stretching region (2800–3000 cm^{-1}), spectroscopic signals consisting of three peaks at 2815, 2905, and 2980 cm^{-1} were observed (Fig. S7-1). The most red-shifted peak, observed well below 2850 cm^{-1}, is specific to the symmetric stretching oscillation of the adsorbed CH_3 species, while the two prominent peaks above 2900 cm^{-1} are also attributable to asymmetric stretching of CH_3 species[14-17]. Therefore, we can assume that methane conversion processes occur at the photocatalyst surfaces and that adsorbed CH_3 species are relatively stable reaction intermediates responsible for the rate-determining step on Pt/Ga_2O_3 photocatalysts. A C–O stretching band (1900–2100 cm^{-1}) was observed as further evidence that multiple reactions of intermediate species (Figs. 5b, S6-1b, and S6-1c) take place on the photocatalyst surface[18].

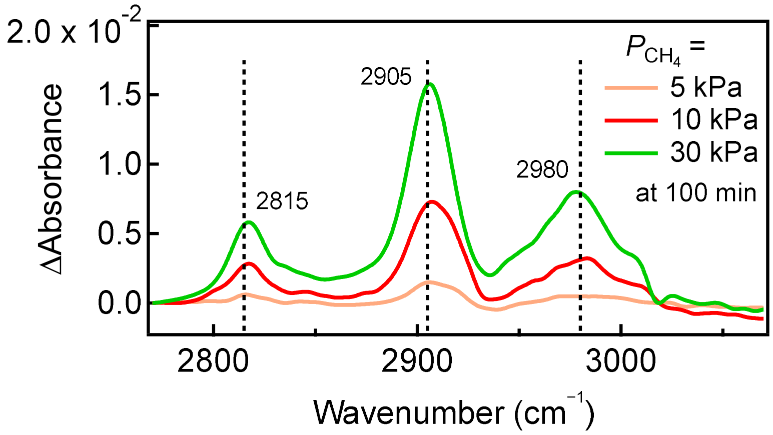

Figure S7-1. O*perando* infrared absorption spectra in the C–H stretching region for Pt/Ga_2O_3 photocatalyst under the steady-state reaction condition at various partial pressure of methane under wet reaction condition (P_{H_2O} = 2 kPa).

Supplementary Note 8

Adsorption features of CH_4 without UV irradiation.

Under photocatalytic reaction conditions, *operando* DRIFT spectroscopy and MD simulations indicated that methane undergoes dissociative adsorption on the photocatalyst surface. To confirm whether such dissociative adsorption is induced only under photocatalytic reaction conditions, we evaluated methane adsorption without UV excitation light using volumetric adsorption measurements and DRIFT spectroscopy. Adsorbed methane molecules were not detected at room temperature without UV irradiation. Therefore, we can conclude that methane molecules cannot chemisorb on surfaces without UV irradiation and that dissociative chemisorption only takes place under UV irradiation.

To further investigate the physisorption properties of methane, we conducted volumetric adsorption measurements of methane at liquid nitrogen temperature (~77 K). Fig. S8-1 shows the amount of methane adsorbed on the catalyst as a function of methane pressure at the liquid N_2 temperature. The adsorbed amount was well fitted by the BET adsorption isotherm equation (equation (*S*1_1)). Based on the E_M = 9.6 kJ/mol for the sublimation enthalpy of methane at 77 K, adsorption energy of methane for the first layer is estimated to be 12–15 kJ/mol for the sample surfaces Pt/Ga_2O_3, $Pt/NaTaO_3$ and Pt/TiO_2. These low E_1 values clearly indicate that methane molecules are only weakly physisorbed on the sample surface without UV irradiation[19,20].

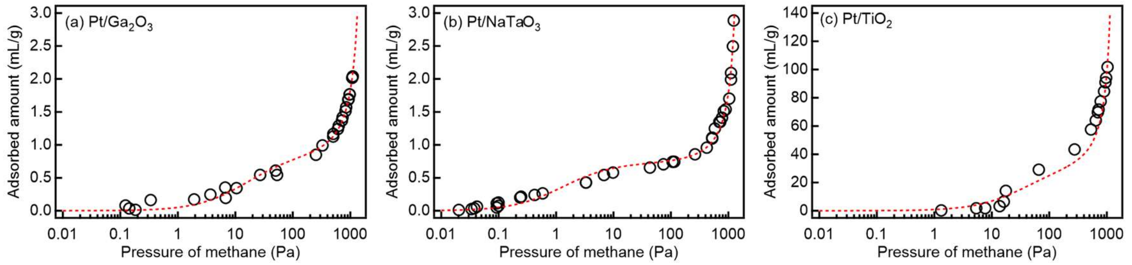

Figure S8-1. Adsorption of methane as a function of methane pressure at liquid N_2 temperature on (a) Pt/Ga_2O_3, (b) $Pt/NaTaO_3$, and (c) Pt/TiO_2 photocatalysts.

Table S8-1. BET fitting parameters of adsorbed methane.

	a (mL/g_{cat})	$E_1 - E_M$ (kJ/mol)	E_1 (kJ/mol) [a]	T_s (K)
Pt/Ga_2O_3	0.84	3.17	12.8	79.7
Pt/$NaTaO_3$	0.73	4.72	14.3	79.2
Pt/TiO_2	30.9	2.42	12.0	78.4

a E_1 was calculated by the following equation: $E_1 = E_M + (E_1 - E_M) = 9.6$ kJ/mol + $(E_1 - E_M)$, with the sublimation enthalpy of methane $E_M \approx 9.6$ kJ/mol at ~77 K and the obtained fitting parameter $E_1 - E_M$.

Supplementary Note 9
Effects of sample heating on photocatalysis.

No appreciable methane conversion occurred by heating of the Pt/Ga_2O_3 photocatalyst to ~318 K with a halogen lamp. Note that the wavelength of this halogen lamp (500–3500 nm) is insufficient for exciting valence band electrons of Ga_2O_3 whose band gap energy is corresponding to ~260 nm. As shown in Fig. S9-1, almost no production derived from methane conversion was detected at ~318 K without UV irradiation while H_2 and CO_2 yields evolved linearly under UV irradiation after the induction period[21–25] of ~5 min. Therefore, the increase in the sample temperature induced by UV irradiation does not have an influence on the photocatalytic C–H activation of methane.

It is also noteworthy that the reaction temperature rose in the 5–20 min under UV irradiation from ~309 K to ~318 K (Fig. S9-1b) while the CO_2 and H_2 yields linearly increased in this period. This indicates that the photocatalytic activity does not depend on the sample temperature within this temperature range.

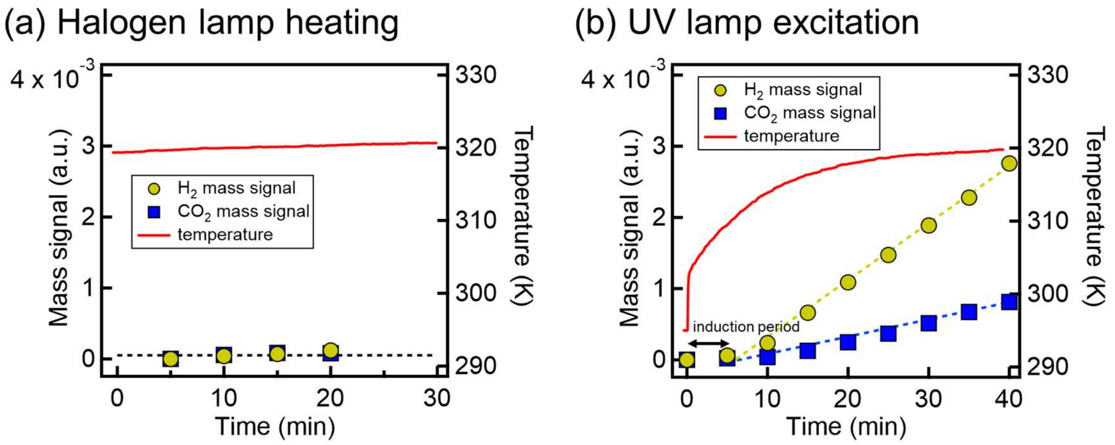

Figure S9-1. Time profiles of the sample temperature and the H_2 and CO_2 mass signal produced on Pt/Ga_2O_3 photocatalysts at a methane partial pressure of 70 kPa and water partial pressures of 2 kPa under (a) only halogen lamp irradiation (thermal heating condition; ~318 K) and (b) UV lamp irradiation. Under the UV irradiation, the products were evolved linearly after the induction period of ~5 min, while almost no change on mass signal was detected under the halogen lamp irradiation.

The sample heating derived from UV irradiation (Fig. S9-1b) also affects the *operando* IR spectra (Fig. 2). As described in our previous study[26], absorbance change signals derived from thermally excited electrons and thermally desorbed water molecules

are also observed on the *operando* IR spectrum measured during the increase in the photocatalyst sample temperature. Therefore, the *operando* IR measurements were started from the time when the temperature increase leveled off in order to carefully extract the response derived from photocatalytic reaction while excluding the spectral change derived from the sample heating. Notably, one layer of adsorbed water molecules covers the sample at the reached temperature of ~318 K as described in Supplementary Note 1, and the photocatalytic activity was evaluated at this period in this study.

Because the time needed for the sample temperature to level off slightly depends on the photocatalyst samples, the starting time for the IR measurement should also be adequately changed depending on the samples. Thus, time zero (t = 0 min) was defined as the starting time for the measurement.

Supplementary References

1. Shirai K, Sugimoto T, Watanabe K, Haruta M, Kurata H, Matsumoto Y. Effect of water adsorption on carrier trapping dynamics at the surface of anatase TiO_2 nanoparticles. *Nano Letters* **16**, 1323-1327 (2016).
2. Brunauer S, Emmett PH, Teller E. Adsorption of Gases in Multimolecular Layers. *Journal of the American Chemical Society* **60**, 309-319 (1938).
3. Manca C, Martin C, Roubin P. Comparative Study of Gas Adsorption on Amorphous Ice: Thermodynamic and Spectroscopic Features of the Adlayer and the Surface. *The Journal of Physical Chemistry B* **107**, 8929-8934 (2003).
4. Kato F, Sugimoto T, Matsumoto Y. Direct Experimental Evidence for Markedly Enhanced Surface Proton Activity Inherent to Water Ice. *The Journal of Physical Chemistry Letters* **11**, 2524-2529 (2020).
5. Li F, Skinner JL. Infrared and Raman line shapes for ice Ih. I. Dilute HOD in H_2O and D_2O. *The Journal of Chemical Physics* **132**, 204505-204505 (2010).
6. Shirai K*, et al.* Water-Assisted Hole Trapping at the Highly Curved Surface of Nano-TiO_2 Photocatalyst. *Journal of the American Chemical Society* **140**, 1415-1422 (2018).
7. Yuliati L, Hattori T, Itoh H, Yoshida H. Photocatalytic nonoxidative coupling of methane on gallium oxide and silica-supported gallium oxide. *Journal of Catalysis* **257**, 396-402 (2008).
8. Singh SP, Anzai A, Kawaharasaki S, Yamamoto A, Yoshida H. Non-oxidative coupling of methane over Pd-loaded gallium oxide photocatalysts in a flow reactor. *Catalysis Today* **375**, 264-272 (2021).
9. Wang G*, et al.* Light-Induced Nonoxidative Coupling of Methane Using Stable Solid Solutions. *Angewandte Chemie International Edition* **60**, 20760-20764 (2021).
10. Bagotzky VS, Vassiliev YB, Khazova OA. Generalized scheme of chemisorption, electrooxidation and electroreduction of simple organic compounds on platinum group metals. *Journal of Electroanalytical Chemistry* **81**, 229-238 (1977).
11. Psofogiannakis G, St-Amant A, Ternan M. Methane oxidation mechanism on Pt(111): A cluster model DFT study. *The Journal of Physical Chemistry B* **110**, 24593-24605 (2006).
12. Nakamura J, Kunimori K, Uchijima T. Measurement and Analysis of Elementary Steps in Catalytic Reactions. *Hyomen Kagaku* **12**, 480-490 (1991).
13. Feng Y, Niiranen J, Gutman D. Kinetic studies of the catalytic oxidation of methane. 2. Methyl radical recombination and ethane formation over 1% strontium/lanthanum sesquioxide. *The Journal of Physical Chemistry* **95**, 6564-6568 (1991).
14. Wolski L, Daturi M, Nowaczyk G, Ziolek M. Insight into methanol photooxidation over mono-(Au, Cu) and bimetallic (AuCu) catalysts supported on niobium pentoxide — An operando-IR study. *Applied Catalysis B, Environmental* **258**, 117978-117978 (2019).

15. Gutiérrez-González A, Beck RD. Quantum state and surface-site-resolved studies of methane chemisorption by vibrational spectroscopies. *Physical Chemistry Chemical Physics* **22**, 17448-17459 (2020).

16. Yoshinobu J, Ogasawara H, Kawai M. Broken symmetry of adsorbed methane and self-limiting photoinduced dissociation on Pt(111). *Surface Science* **363**, 234-239 (1996).

17. Panayotov DA, Burrows SP, Morris JR. Photooxidation Mechanism of Methanol on Rutile TiO_2 Nanoparticles. *The Journal of Physical Chemistry C* **116**, 6623-6635 (2012).

18. Saito H, Sato H, Higashi T, Sugimoto T. (submitted).

19. Yoshinobu J, Ogasawara H, Kawai M. Symmetry Controlled Surface Photochemistry of Methane on Pt(111). *Physical Review Letters* **75**, 2176-2179 (1995).

20. Arumainayagam CR, McMaster MC, Schoofs GR, Madix RJ. Dynamics of molecular CH_4 adsorption on Pt(111). *Surface Science* **222**, 213-246 (1989).

21. An induction period is the initial slow phase of a chemical reaction and is often observed before the reaction system reaches quasi steady-state[22]. Existance of induction periods was often observed on photocatalysis, and the photocatalytic activity was typically evaluated under the quasi steady-state conditions after the induction periods[23–25].

22. Tan P. Active phase, catalytic activity, and induction period of Fe/zeolite material in nonoxidative aromatization of methane. *Journal of Catalysis* **338**, 21-29 (2016).

23. Ebina Y, Sakai N, Sasaki T. Photocatalyst of Lamellar Aggregates of RuO_x-Loaded Perovskite Nanosheets for Overall Water Splitting. *The Journal of Physical Chemistry B* **109**, 17212-17216 (2005).

24. Yoshida H*, et al.* Hydrogen Production from Methane and Water on Platinum Loaded Titanium Oxide Photocatalysts. *The Journal of Physical Chemistry C* **112**, 5542-5551 (2008).

25. Bahruji H*, et al.* Rutile TiO2–Pd Photocatalysts for Hydrogen Gas Production from Methanol Reforming. *Topics in Catalysis* **58**, 70-76 (2015).

26. Sato H, Sugimoto T. *Operando* FT-IR Spectroscopy of Steam-methane-reforming Photocatalyst under Irradiation of Intensity Modulated UV Light. *Vacuum and Surface Science* **63**, 476-481 (2020).
